# N-Doping/KOH Synergy in Waste Moss Biochar for Geosmin Removal in Aquaculture Water: Elucidating Surface Functionalization and Activation Mechanisms

**DOI:** 10.3390/biology14081045

**Published:** 2025-08-14

**Authors:** Zhonghua Li, Xi Chen, Liping Qiu, Huimin Xu, Limin Fan, Shunlong Meng, Zhongquan Jiang, Chao Song

**Affiliations:** 1 Wuxi Fisheries College, Nanjing Agricultural University, Wuxi 214081, China; 18277631081@163.com (Z.L.); fanlm@ffrc.cn (L.F.); mengsl@ffrc.cn (S.M.); 2 Freshwater Fisheries Research Center, Chinese Academy of Fishery Sciences, Wuxi 214081, China; chenxi@ffrc.cn (X.C.); qiulp@ffrc.cn (L.Q.); xuhuimin@ffrc.cn (H.X.); 3 Laboratory of Quality & Safety Risk Assessment for Aquatic Products on Environmental Factors (Wuxi), Ministry of Agriculture and Rural Affairs, Wuxi 214081, China; 4 Key Laboratory of Control of Quality and Safety for Aquatic Products, Ministry of Agriculture and Rural Affairs, Beijing 100141, China; 5 Key Laboratory of Environmental Health Impact Assessment of Emerging Contaminants, Ministry of Ecology and Environment, School of Environmental Science and Engineering, Shanghai Jiao Tong University, Shanghai 200240, China; jiangzq@ecsf.ac.cn; 6 East China Sea Fisheries Research Institute, Chinese Academy of Fishery Sciences, Shanghai 200090, China

**Keywords:** earthy substance, aquaculture water remediation, N-doped biochar, alkali activation, bioresources recycling

## Abstract

Effective removal of geosmin (GSM) from water is crucial to ensuring high-quality drinking water and sustainable aquaculture worldwide. In this study, response surface methodology with a central composite design optimized the synthesis of moss-derived, nitrogen-doped biochar (KNBC) via ammonia hydrothermal treatment (AHT) coupled with KOH activation, and its performance was compared to the non-activated AHT biochar (NBC). Textural analysis revealed that KNBC exhibited a higher specific surface area (62.13 m^2^/g) than NBC (57.05 m^2^/g) and the virgin biochar (BC, 18.55 m^2^/g). KOH activation markedly expanded mesopore volume and diameter, enhancing GSM mass transfer. KNBC demonstrated the highest adsorption capacity (3.933 µg/g), which was not significantly different from that of NBC (3.807 µg/g) but 67.33-fold of that of virgin biochar. Kinetic data were best described by the pseudo-second-order model, while equilibrium isotherms fitted both Langmuir and Sips models, consistent with heterogeneous monolayer adsorption. N-doping was found to modulate the electronic and physicochemical microenvironment of active sites. GSM removal was driven by synergistic hydrophobic interactions, hydrogen bonding, and weak electrostatic attractions at N-/O-containing functionalities. Density functional theory calculations identified pyrrolic-N and graphitic-N functionalities as principal adsorption centers. Furthermore, KNBC retained about 90% of its capacity after four regeneration cycles and proved effective in both synthetic and real water matrices. These findings indicate the potential of moss-based N-doped hydrochar as efficient, reusable adsorbents for GSM removal in drinking water and aquaculture applications.

## Simple Summary

Geosmin (GSM), a major contributor to taste and odor (T&O) issues in water, is challenging to remove using conventional methods. Our study developed nitrogen-doped biochar (KNBC) derived from mosses produced by aquatic eutrophication through ammonia heat treatment and KOH activation techniques. The synthesis process was optimized using response surface methodology with a central composite design. This study presents a novel and sustainable approach for addressing the critical issue of T&O compounds in aquaculture systems by developing high-performance algal-based adsorbents.

## 1. Introduction

Taste and odor (T&O) has always been a critical issue in drinking water treatment and quality improvement of aquatic products [1,2]. Geosmin (GSM), a volatile bicyclic terpene, is frequently encountered in eutrophic waters with a low threshold concentration (4 ng/L) and relatively high hydrophobicity [3]. Due to its small molecular weight (182.3 g/mol) and trace levels (tens to hundreds ng/L) in the aqueous environment, GSM is difficult to remove effectively by conventional flocculation, sedimentation, and filtration methods. Furthermore, typical oxidative processes (chlorination, ozonation, etc.) are not sufficient to efficiently remove GSM from contaminated drinking and aquaculture water [4]. Currently, numerous strategies have been developed for the removal of GSM, including laboratory studies [4,5,6,7,8] and applications in real-scale applications [9,10].

Adsorption has garnered considerable attention as an alternative strategy for mitigating T&O issues, owing to its operational simplicity, cost-effectiveness, and high removal efficiency. Powdered activated carbon (PAC) remains the benchmark adsorbent for GSM removal; however, its application is hampered by limited regenerability, the complexity of activation procedures, and relatively high production costs [5,11]. In recent decades, research has shifted toward low-cost, sustainable adsorbents derived from natural biomass or agricultural by-products [12]. More specifically, biochar-based adsorbents produced from aquaculture residues, such as harmful green algae (e.g., moss), offer dual benefits of waste valorization and T&O control, with potential applicability across diverse water matrices. To date, only a handful of studies have explored aquaculture by-products for water-pollutant removal [13,14]. In our previous work, we synthesized various biochar from three distinct aquaculture wastes and demonstrated that moss-based biochar exhibited the highest GSM uptake; nevertheless, its adsorption capacity (58.41 ng/g) was constrained by intrinsic pore-structure and surface-chemistry limitations, and it showed insufficient removal of co-occurring natural organic matter [14]. Likewise, Antonopoulou et al. [15] reported a spirulina-derived carbon material with the GSM capacity of only 2.353 μg/g. To overcome the limitations of virgin biochar, various enhancement strategies, such as hydrothermal treatment, targeted functional-group incorporation, defect engineering, and composite formation, have been proposed to tailor textural and chemical properties for improved adsorption performance.

Physical and chemical activation protocols are well established for markedly enhancing the specific surface area (SSA) and porosity of biochar. In particular, chemical activation with alkaline agents (e.g., NaOH and KOH) typically yields more extensively developed pore networks and higher SSA than physical treatments [16]. In parallel, heteroatom doping (e.g., N, S, and P) offers a complementary strategy to tailor surface chemistry by introducing new active sites and structural defects, thereby further augmenting adsorption capacity [17]. Among potential dopants, nitrogen is especially compatible with the carbon lattice owing to its similar atomic radius, facilitating facile substitution of C by N within the sp^2^ carbon framework. N-doping not only generates additional π-electrons but also modulates electronic density, strengthening interactions between GSM molecules and the carbon matrix and thus improving removal efficiency [18]. Conventionally, N-doped biochar is produced via co-pyrolysis of biomass with external nitrogen sources (e.g., ammonia and urea), during which sequential reactions, direct cyclization, dehydration, decarbonylation, decarboxylation, and Maillard reactions incorporate N into the carbon network [19]. Aqueous ammonia (NH_3_·H_2_O) is among the most widely used nitrogen precursors. For example, Feng, et al. [20] combined ammonia hydrothermal treatment with chemical activation to generate hierarchical N-functionalized pore structures that demonstrate excellent phenol adsorption, while Mao et al. [16] employed ammonia wet treatment coupled with alkali activation to prepare N-doped hierarchical biochar with ultrahigh SSA for efficient dye removal. Given GSM’s relative hydrophobicity, N-doping not only promotes the formation of O- and N-containing functionalities conducive to hydrophobic and π–π interactions but, when paired with chemical activation, also enhances SSA, pore-size distribution, and mass-transfer kinetics, thereby improving accessibility to adsorption sites.

Ammonia hydrothermal treatment (AHT) coupled with chemical activation was employed to engineer hierarchical, function-rich architectures within moss-derived biochar. First, an ammonia hydrothermal process introduced abundant O- and N-containing moieties into the biomass while simultaneously enhancing pore development and graphitization. Subsequent KOH activation produced biochar characterized by high specific surface area, well-defined porosity, and enriched surface functionality. Response surface methodology (RSM) was then used to optimize synthesis parameters, yielding an N-doped biochar (KNBC) with maximal GSM adsorption capacity. The non-activated AHT biochar (NBC) was synthesized in parallel to isolate and evaluate the specific contributions of KOH activation. Both materials underwent comprehensive characterization, including elemental analysis, spectroscopic identification of functional groups, and electron microscopy of microstructural features, and their adsorption behavior was probed via kinetic, isotherm, and thermodynamic studies. KNBC demonstrated excellent recyclability and practical efficacy in model and real water matrices. Finally, density functional theory (DFT) calculations pinpointed the principal adsorption sites responsible for GSM capture. This work establishes a novel, sustainable strategy for the fabrication of high-performance, bio-based adsorbents for aquatic T&O compounds remediation.

## 2. Materials and Methods

### 2.1. Chemicals and Reagents

All reagents and solvents employed in this study were of analytical grade. NH_3_·H_2_O, KOH, HCl, methanol, and sodium chloride were purchased from ANPEL Laboratory Technologies (Shanghai) Inc., Shanghai, China. Geosmin (GSM, ≥97% purity, 10 mg) and its calibration standard solution (≥98% purity, 100 µg/mL in methanol) were purchased from Sigma-Aldrich (Saint Louis, MO, USA) and o2si Smart Solutions (North Charleston, SC, USA), respectively. The stock standard was diluted in methanol to 1 µg/mL and stored at −20 °C in the dark until use. Ultrapure water produced by a Master-QUT purification system from HHitech (Shanghai, China) was used throughout all experimental procedures.

### 2.2. Preparation of the N-Doped Hydrochar

The synthesis of N-doped hydrochar was systematically optimized through response surface methodology (RSM), which harnessed a central composite design to model three critical parameters—biomass-to-nitrogen ratio (B/N: 1–3), hydrothermal temperature (HT: 150–250 °C), and pyrolysis temperature (PT: 400–800 °C)—with minimal experimental iterations. This RSM-driven optimization decoded multifactorial synergies to maximize GSM adsorption capacity.

Aquaculture-harvested moss biomass was dehydrated (105 °C, 24 h), milled, and sieved (<150 μm). Nitrogen doping was engineered via ammonia hydrothermal treatment (AHT): 5 g biomass in 25 g/L NH_3_·H_2_O underwent pressurized heating (150–250 °C, 1 h) in a stainless-steel reactor. The resultant hydrochar was pyrolyzed under N_2_ (10 °C/min ramp to 400–800 °C; 2 h dwell) and chemically activated with KOH (1:2 mass ratio) through sequential stirring (60 °C, 2 h), acid washing (0.1 M HCl), and vacuum drying (85 °C, 6 h) to yield KOH-activated N-doped hydrochar (KNBC).

**Figure 1:**
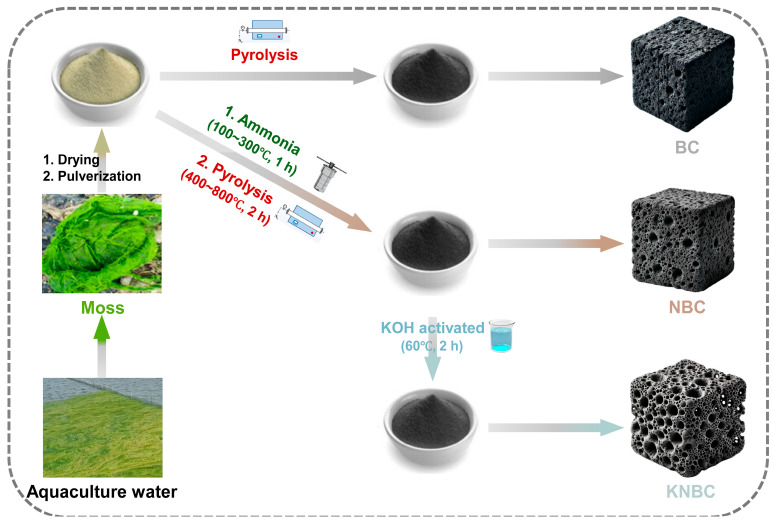
Process diagram for preparing BCs from aquaculture moss raw materials. BC: unmodified moss biochar, NBC: N-doped moss biochar, and KNBC: KOH-activated N-doped moss biochar.

RSM-optimized KNBC (Table S1) was selected for GSM adsorption studies alongside two controls: (1) non-activated NBC (AHT + pyrolysis only) and (2) virgin biochar (BC) from direct pyrolysis (Figure 1).

### 2.3. Characterization Methods for Biochar

All the detailed characterization instrument conditions are included in the Supplementary Information (Text S1).

### 2.4. Adsorption Experiments of GSM on Hydrochar

Batch adsorption experiments were conducted at 25 °C in 250 mL conical flasks, each containing 200 mL of geosmin solution (1000 ng/L). Flasks were agitated at 180 rpm for 120 min in a thermostatic shaker, and all assays were performed in triplicate to ensure reproducibility. To determine the optimal biochar dosage, NBC loadings of 50–300 mg/L were evaluated. The effect of solution pH (3.0–9.0) on adsorption was examined by adjustment with 0.1 mM HCl or NaOH. The influence of dissolved organic matter was probed by spiking humic acid (HA) at concentrations of 0.01–20 mg/L. Upon completion of adsorption, suspensions were filtered through 0.45 µm organic-phase syringe filters, and residual geosmin concentrations were quantified by gas chromatography–mass spectrometry (GC–MS, see Text S2 for instrumental parameters).

Adsorption kinetics were assessed by sampling at predetermined intervals from 0 to 180 min. The equilibrium isotherms were obtained using initial geosmin concentrations ranging from 200 to 1200 ng/L. In the thermodynamic experiments, parameters (Δ*G°*, Δ*H°*, and Δ*S°*) were derived from isotherm data collected at four temperatures (15, 25, 35, and 45 °C).

Reusability tests were conducted by recovering used NBC and KNBC (NBCs) via filter membrane, desorbing in various eluents for 12 h, rinsing with deionized water, and drying at 60 °C prior to reuse in subsequent cycles. To evaluate practical applicability, adsorption experiments were also performed using GSM-contaminated pond and lake water samples.

### 2.5. Data Processing and Statistical Analysis

The kinetic, isotherm, and thermodynamic models applied in this study are detailed in Text S3. Density functional theory (DFT) calculations were conducted using Gaussian 16, with computational parameters and methodological specifics provided in Text S4 [21,22]. Data visualization, model fitting, and statistical analyses were performed using Origin 2024.

## 3. Results and Discussion

### 3.1. RSM for Selecting the Optimal KNBC

**Figure 2:**
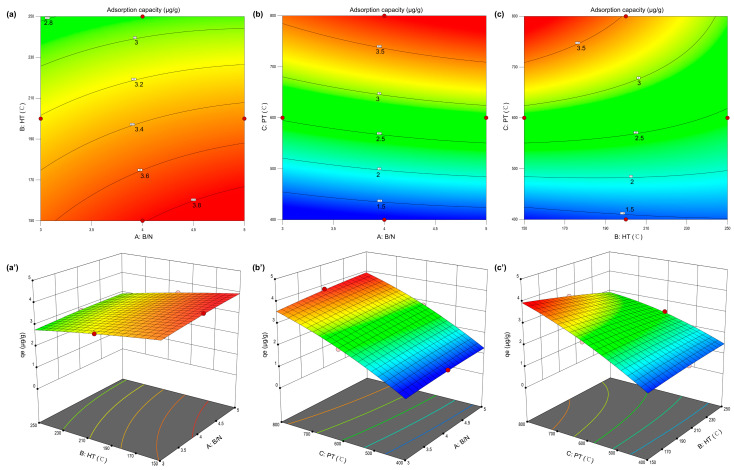
Response surface methodology to optimize adsorption experiments, detailed on 2D and 3D response surface plots. (**a**,**a’**) B/N and HT, (**b**,**b’**) B/N and PT, and (**c**,**c’**) HT and PT of adsorption capacity for geosmin response 2D and 3D plots.

RSM was employed based on Box–Behnken Design (BBD), using Design Expert 13 software, with results showed in Figure 2. In the one-factor preliminary experiment, we successfully identified the optimal ranges for the biomass-to-nitrogen ratio (B/N, 3–5) and hydrothermal temperature (HT, 150–250 °C), with details presented in Figure S1 and Table S1.

**Figure 3:**
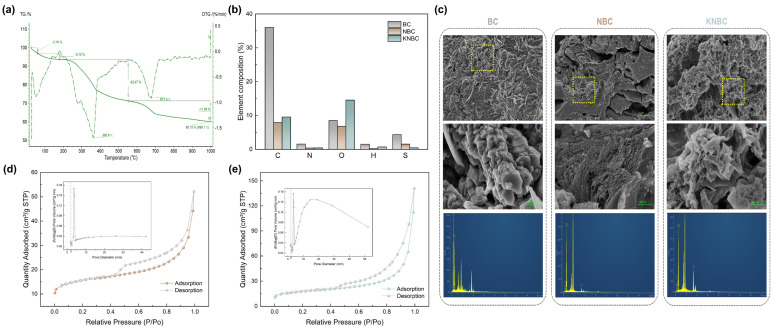
Optimization of biochar pyrolysis conditions and characterization of modified biochar. (**a**) TG/DTG curves of dry moss powder. (**b**) Elemental analysis. (**c**) SEM map and EDS image, and the N_2_ adsorption–desorption isotherms (insert: the pore-size distribution curves) of the N-doped hierarchical biochar ((**d**): NBC, (**e**): KNBC).

Thermogravimetric analysis (TGA) of oven-dried moss powder (Figure 3a) delineated three principal thermal regimes. An initial mass loss of 6.29% from 30 to 171.7 °C was attributed to moisture desorption. The primary decomposition phase, spanning 171.7 to 780.5 °C, accounted for 30.92% mass loss and encompassed cellulose dehydration between 300 and 400 °C, with the maximum rate of weight loss occurring at 362.6 °C (DTG peak). Beyond 667 °C, graphitization of the carbon matrix became dominant, yielding a residual mass of 62.79% at 780.5 °C and 60.15% at 1000 °C. Based on these thermal transitions, pyrolysis temperatures of 400, 600, and 800 °C were selected for the preparation of moss-derived biochar [23].

The factor levels and coded variables are presented in Tables S1 and S2. A total of 17 randomized experimental runs, including five replicates at the central point, were performed to assess the individual and interactive effects of B/N (A), HT (B), and PT (C) on GSM adsorption by KNBCs (Table S3). Three-dimensional response surface and contour plots are shown in Figure 2, and the results of ANOVA and lack-of-fit tests are summarized in Tables S4 and S5. From these data, the quadratic polynomial equation (real factors) relating the three synthesis parameters to adsorption capacity was derived as follows:

(1) $$\begin{array}{c} q_{e}=-7.58253+0.520050A+0.026641B+0.017047C-\\ 0.001155\mathrm{AB}+0.000184\mathrm{AC}-0.000030\mathrm{BC}-\\ 0.037725A^{2}-0.000017B^{2}-\left(5.64313\times 10^{-6}\right)C^{2} \end{array}$$

Based on the *p*-value, the significant factors influencing the model were identified, and the above equation was optimized as the following:

(2) $$\begin{array}{c} q_{e}=-7.58253+0.520050A+0.026641B+0.017047C\\ -0.000030\mathrm{BC}-\left(5.64313\times 10^{-6}\right)C^{2} \end{array}$$

Presented in Table S4, the analysis of variance (ANOVA) for the quadratic regression model details critical statistical metrics: sum of squares (SS), degrees of freedom (df), mean square (MS), F-statistic, and the associated *p*-value. The model exhibited a high F-value of 258.58, indicating the overall significance of the regression model. Given that the associated *p*-value was below 0.05, the probability that such a large F-value could result from random noise is less than 0.01%, confirming the statistical robustness of the model. Furthermore, the lack-of-fit F-value of 0.9417 suggests that the lack of fit is not significant relative to the pure error, thereby supporting the model’s adequacy [24]. The model terms with *p*-values less than 0.05, A, B, C, BC, and C^2^, were identified as statistically significant. The magnitude of the corresponding F-values reflects the relative influence of these variables on the adsorption response. In descending order, the PT (C, F = 2093.65) exhibited the most significant effect, followed by HT (B, F = 45.94) and B/N (A, F = 20.35). Therefore, PT was identified as the most influential factor in enhancing adsorption capacity, suggesting its critical importance for practical optimization of the adsorbent. PT was the master switch for geosmin adsorption, simultaneously sculpting micropores, stripping oxygen to heighten hydrophobicity, and preserving π-electron-rich turbostratic carbon. Conversely, the relatively lower influence of biomass-to-nitrogen ratio implies that the adsorbent performance remains stable across a broad range of NH_3_·H_2_O dosages. The model’s reliability was further validated by its statistical indicators: the coefficient of determination (*R*^2^ = 0.997), adjusted *R*^2^ (*R*^2^*_adj_* = 0.9931), and predicted *R*^2^ (*R*^2^*_pre_* = 0.9774), as detailed in Table S5. These values confirm the model’s high predictive power and minimal deviation from experimental results. Moreover, the signal-to-noise ratio of 55.12, substantially exceeding the threshold value of 4, further affirms the model’s precision and applicability. Collectively, these results demonstrate that the proposed model is statistically valid and suitable for use as a predictive tool within the defined experimental design space.

Response surface analysis was performed using Design-Expert 13 software. By fixing the levels of one variable and varying the remaining two, three-dimensional response surface and contour plots were generated to visualize the interaction effects between factors. Figure 2 illustrates the interactions between B/N and the other two parameters. As shown in Figure 2a,a’, the range of material adsorption capacity was smaller when B/N and HT interacted, indicating that the influence of these two factors was relatively minor. However, as shown in Figure 2b,c, when PT was present, the range of material adsorption capacity increased from 1.5 to 3.5 μg/g, indicating that PT was the most critical factor influencing the adsorption capacity of nitrogen-doped biochar. HT has a greater influence on adsorption capacity than B/N, as the red area in Figure 2c,c’ is smaller and more curved. The optimal adsorption capacity was observed at a B/N ratio of approximately 4.5–5.0, indicating that excessive N-doping may not yield further improvements in performance. Additionally, pyrolysis temperature exerted the most pronounced effect on the equilibrium adsorption capacity, while the other parameters exhibited a more linear relationship with adsorption performance. Furthermore, it was possible to visually observe the adsorption capacity of KNBCs for GSM under any given conditions within the experimental space. This significantly enhances the potential for further applications and research on KNBCs. Based on the model prediction, the maximum adsorption capacity of 3.946 μg/g was achieved under the optimized conditions of B/N = 5, HT = 150 °C, and PT = 800 °C. Experimental verification under these conditions yielded an adsorption capacity of 3.933 μg/g, demonstrating the high accuracy and reliability of the model.

### 3.2. Characterization of Biochar

#### 3.2.1. Elemental Analysis

Elemental mapping and quantitative analysis reveal pronounced compositional shifts among moss-derived BC, NBC, and KNBC, driven by torrefaction and subsequent treatments (Figure 3b and Table S7). C content plummeted from 36.71% (BC) to 7.94% (NBC) and 9.52% (KNBC), attributed to hydrothermal carbon volatilization and structural fragmentation. Oxygen dynamics followed distinct trajectories: NBC exhibited O depletion (15.82% → 9.17%) via thermal deoxygenation, whereas KNBC regained oxygenicity (11.35%) through KOH-derived oxygenic functional groups. N content decreased from 1.53% (BC) to 0.37% (NBC) and 0.40% (KNBC), reflecting volatilization of moss-inherent N-compounds during AHT. BC was prepared directly from moss through a single-step pyrolysis process, while NBC was produced by hydrothermal treatment of moss and ammonia water in a reactor at 150 °C, followed by pyrolysis. During the hydrothermal process, a large amount of ammonia gas was generated and evaporated, further consuming the nitrogen in moss, resulting in a reduced nitrogen percentage in the final NBC. Partial N-retention arose from competitive Maillard reactions between NH_3_-derived radicals (NH_2_*/NH*) and oxygenic moieties (-COOH, -C=O, -OH) [25,26], while thermal deoxygenation pathways liberated volatile oxygen as H_2_O, CO, and CO_2_ [27]. KNBC’s optimized C-O-N equilibrium, synergized with hierarchical porosity, positions it as a superior N-doped biochar synthesized via ammonia-rich torrefaction and controlled KOH activation, a strategy that balances elemental tailoring and structural stability for enhanced adsorption performance.

#### 3.2.2. Textural Properties

Figure 3c compares the surface morphologies of pristine BC, NBC, and KNBC. Virgin BC displayed a coarse, minimally porous surface dominated by blocked, irregularly distributed monolayered pores. Its pore architecture consisted of fragmented thick lamellae, a morphology likely linked to the lack of torrefaction pretreatment. In contrast, NBC (synthesized via AHT) exhibited a smoother yet pore-dense surface with heterogeneous pore sizes. Notably, KNBC showcased a three-dimensional hierarchical porosity featuring enlarged, well-defined pores, a direct consequence of KOH activation. Here, KOH acted as a chemical etcher, removing pore-blocking residues and amplifying porosity through structural etching [28]. Despite these morphological enhancements, KNBC’s specific surface area (SSA, 62.13 m^2^/g) showed only a marginal increase over NBC (57.05 m^2^/g, Table 1), suggesting a trade-off between KOH-induced pore expansion and partial structural collapse. EDS analysis further confirmed enhanced K^+^ retention in KNBC, verifying successful KOH incorporation and its reactive interplay with the carbon framework.

The N_2_ adsorption–desorption isotherms and pore-size distributions of NBCs are displayed in Figure 3d,e. All samples showed dual adsorption regimes: (1) low-pressure monolayer adsorption (P/P_0_ < 0.1) within micropores and (2) high-pressure capillary condensation (0.45 < P/P_0_ < 0.99) in mesopores. The isotherms align with International Union of Pure and Applied Chemistry (IUPAC) type I/IV profiles featuring H4 hysteresis loops, confirming slit-shaped micro-mesopore coexistence. Notably, pore-size analysis revealed hierarchical micro-mesoporous architectures in N-doped biochar, with KNBC demonstrating optimal structural ordering. This dual-scale porosity enhances organic pollutant adsorption by synergistically improving accessible surface area and mass transport kinetics [29,30].

As evidenced by the pore-size distribution profiles in Figure 3d,e, all NBCs exhibited bimodal micropore maxima at 1.7 and 1.9 nm. KNBC distinctly outperformed NBC in mesopore abundance (2–30 nm range), with structural parameters quantitatively compared in Table 1. N-doping universally enhanced porosity metrics: both NBC and KNBC showed significant increases in BET-specific surface area (SSA), total pore volume, and micro-/mesopore surface areas/volumes. While their BET SSA and micropore volumes were comparable, KNBC achieved superior Langmuir SSA (37.179 m^2^/g mesoporous surface area; 0.206 cm^3^/g mesoporous volume), constituting 94.50% of its total pore volume. These metrics align with KNBC’s pronounced hysteresis loops and mesopore peaks (Figure 3d,e), confirming its optimized mesopore dominance. The hierarchical mesoporosity arises synergistically from (1) moss biomass-derived precursors and ammonia-mediated crosslinking during hydrothermal treatment and (2) KOH’s selective etching of carbon matrices to expand pore networks. However, excessive KOH dosage risks structural over-etching, as evidenced by localized pore collapse in KNBC’s SEM morphology (Figure 3c), underscoring the necessity of balanced activation conditions.

Consistent with the previous literature, the formation of microporous and mesoporous structures in biochar is primarily attributed to the thermal degradation of lignin and cellulose, respectively [31]. The enhanced mesoporosity observed in moss-derived biochar (Table 1) is largely attributed to the effective removal of hemicellulose and cellulose during the torrefaction process. The resulting N-doped biochar, with its larger SSA and hierarchical micro-mesoporous structure, provides an ideal matrix for the incorporation of N- and O-containing functional groups, which are essential for efficient GSM adsorption [32]. Micropores contribute significantly to adsorption affinity through strong surface interactions, while mesopores reduce mass transfer resistance and facilitate rapid diffusion of GSM molecules. This synergistic micro-mesoporous architecture enhances the adsorption performance of N-doped hydrochar, making KNBC a promising adsorbent for practical applications.

#### 3.2.3. Evolution of the Functional Groups and Properties

**Figure 4:**
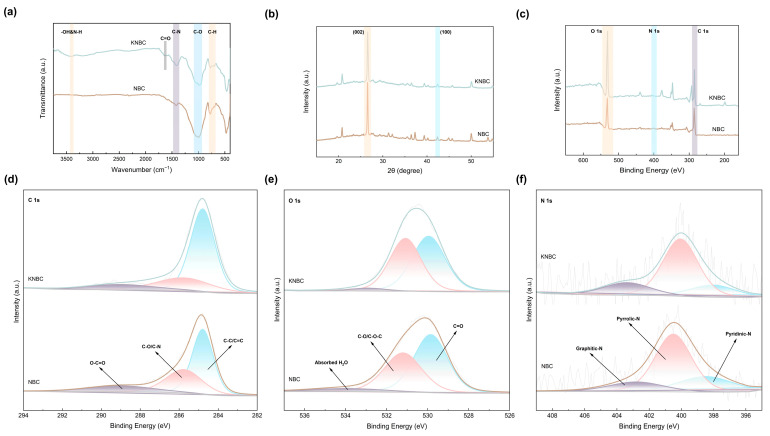
(**a**) The FT-IR spectra, (**b**) XRD patterns, and (**c**) XPS analysis of NBC and KNBC. (**d**) C 1s, (**e**) O 1s, and (**f**) N 1s.

FT-IR analysis (Figure 4a) tracks the nitrogen-driven evolution of surface functionalities in moss-derived biochar. All samples exhibited characteristic bands at 3405 cm^−1^ (-OH/-NH stretching), 1420–1250 cm^−1^ (C-N), 1125–990 cm^−1^ (C-O), and 800–650 cm^−1^ C-H) [16]. Strikingly, KNBC uniquely displayed a C=O stretching signature at 1618 cm^−1^, absent in other variants. Relative to pristine BC [13], NBC and KNBC showed attenuated -OH/-NH, C=O, and C-O intensities, confirming torrefaction-induced deoxygenation. Conversely, ammonia-assisted treatment amplified the 3405 cm^−1^ (-OH/-NH) and 1420–1250 cm^−1^ (C-N) absorptions—direct spectral evidence of nitrogen integration into the carbon lattice [16]. This N-functionalization intensified in KNBC, where KOH activation synergistically stabilized N-containing groups through enhanced surface reactivity and structural reorganization.

The crystalline structure and chemical composition of the N-doped hierarchical biochar were further elucidated via X-ray diffraction (XRD) and X-ray photoelectron spectroscopy (XPS). In the XRD patterns (Figure 4b), a broad diffraction peak centered around 26° was attributed to the (002) plane of amorphous carbon, while a weaker peak near 42° corresponded to the (100) plane of graphitic carbon [33], confirming the predominance of a disordered carbon structure in the synthesized biochar. The sharper (002) peak in the AHT-treated samples suggests a slight increase in crystallinity due to the rearrangement of carbon layers induced by hydrothermal conditions. Moreover, both (002) and (100) reflections in KNBC exhibited higher intensities than those in NBC, indicating that KOH activation enhanced the degree of graphitization or increased the proportion of these crystal facets within the carbon framework [34]. The relatively broad and smooth peak near 42° in all samples can be ascribed to the lattice distortion and disorder introduced by N-doping, which disrupts the regular stacking of graphitic layers [35].

XPS analysis (Figure 4c) validates ammonia-mediated nitrogen doping in biochar, with a distinct N 1s peak at 400.0 eV confirming N integration via hydrothermal treatment. Deconvolution of C 1s spectra (Figure 4e) identifies three carbon states: graphitic C–C/C=C (284.8 eV), oxygen/nitrogen-bonded C–O/C–N (285.8 eV), and carboxyl O–C=O (289.0 eV) [36]. Strikingly, KNBC’s graphitic carbon fraction (66.03% C–C/C=C) surpasses NBC (52.91%, Figure S5), highlighting KOH-enhanced graphitization. The C–O/C–N moieties, critical electron-transfer mediators via π–π interactions [37], originate from hydroxyl groups and lattice-incorporated nitrogen.

O 1s spectral resolution (Figure 4e) further reveals three oxygen states: C=O (529.9 eV), hydroxyls (531.2 eV), and adsorbed H_2_O (534.0 eV) [23], collectively enabling hydrogen-bond-driven GSM adsorption. Nitrogen speciation analysis (Figure 4f) tracks transformative doping dynamics: Maillard reactions between moss-derived oxygenic groups and NH_3_-derived radicals (NH_2_*/NH*) that yield pyridinic-N, pyrrolic-N, and graphitic-N [38]. While pyrrolic-N dominates NBC (61.11%), KOH activation redistributes nitrogen into pyridinic-N and graphitic-N, thermodynamically favored configurations that amplify adsorption through electron-donating moieties and targeted pollutant interactions [39].

### 3.3. GSM Adsorption Ability of N-Doped Hydrochar

#### 3.3.1. Effect of the Hydrochar Content

**Figure 5:**
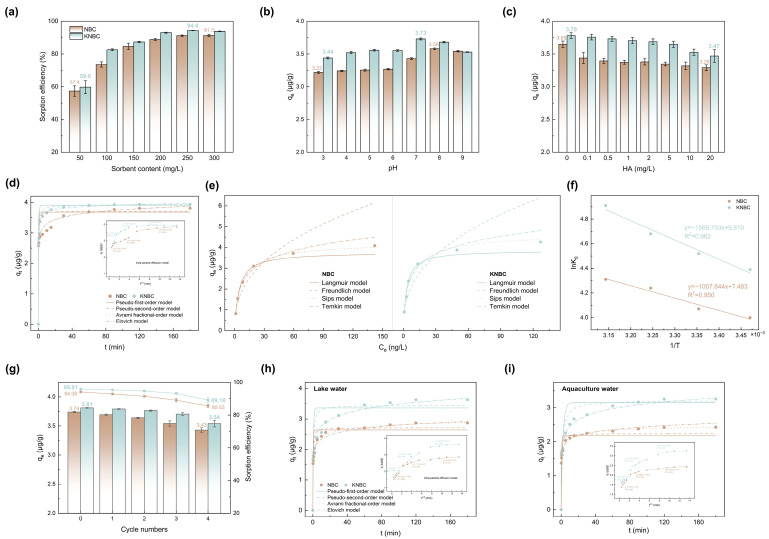
Effect of (**a**) NBCs’ dosage and (**b**) pH and (**c**) humic acid (HA) on geosmin adsorption by NBC and KNBC. (**d**) Geosmin adsorption kinetics, (**e**) the equilibrium adsorption isotherms, and (**f**) the adsorption thermodynamics of NBC and KNBC in aqueous solution at pH 7.0. (**g**) Reusability of NBC and KNBC. (**h**,**i**) The adsorption performance of NBC and KNBC in real water application. (Experiment conditions: [geosmin]_0_ = 1 μg·L^−1^, V = 0.2 L, [NBCs]_0_ = 0.25 g·L^−1^, pH = 7.0 ± 0.1, T = 25 °C, t = 120 min).

As shown in Figure 5a and Figure S2, GSM removal efficiency increased with the dosage of hydrochar, while the adsorption capacity (*q_e_*) decreased. The higher removal at larger dosages is attributed to the greater number of available adsorption sites. However, since the initial GSM concentration remained constant, *q_e_* decreased due to the non-linear relationship between adsorbent mass and adsorption capacity. At pH 7.0 and an initial GSM concentration of 1 μg/L, KNBC achieved a maximum removal efficiency of 94.4% at 250 mg/L, whereas NBC required over 300 mg/L to reach similar performance (Figure 5a). This may be due to the higher SSA of KNBC, as SSA and porosity significantly influence adsorption. Given that GSM concentrations below 10 ng/L can cause earthy odors in drinking water and aquatic products [1,40], minimizing residual GSM is essential. In this study, 250 mg/L was identified as the optimal dosage, achieving 94.4% removal. A further increase to 300 mg/L slightly reduced efficiency (93.9%), possibly due to particle aggregation. Therefore, 250 mg/L was used in subsequent experiments.

#### 3.3.2. Effect of pH and Natural Organic Matter

The pH of the GSM solution significantly influences adsorption due to the ionization state of GSM and the surface functionalities of biochar. Adsorption performance was evaluated across a pH range of 3.0–9.0 (Figure 5b). For KNBC, the adsorption capacity increased from 3.44 μg/g at pH 3.0 to a maximum of 3.73 μg/g at pH 7.0, then decreased at higher pH values. In contrast, NBC exhibited its highest adsorption capacity of 3.58 μg/g at pH 8.0. The activation of KOH enabled the hydrochar to have a wide pH tolerance range in water. Near neutral pH, the hydrochar surface approached electroneutrality, leading to minimal electrostatic interaction with partially dissociated GSM (-O^−^). At pH > 7, deprotonation of O- and N-containing groups imparted a negative charge to the hydrochar surface. As GSM was also negatively charged under these conditions, electrostatic repulsion occurs, inhibiting adsorption [41]. Based on these results, subsequent adsorption experiments were conducted at pH 7.0 using deionized water.

#### 3.3.3. Adsorption Kinetics

The adsorption kinetics of GSM on NBCs were investigated, with results shown in Figure 5d. GSM uptake increased rapidly within the first 10 min, likely due to the abundance of available adsorption sites. Removal efficiencies reached 76.15% for NBC and 91.15% for KNBC, with equilibrium attained within 120 min and corresponding adsorption capacities of 3.807 μg/g and 3.933 μg/g, respectively. To elucidate the adsorption mechanism, the kinetic data were fitted to five models: pseudo-first-order, pseudo-second-order, Avrami fractional order, Elovich, and intra-particle diffusion (Text S3, Equations (S3)–(S7)) [42].

Based on higher coefficients (*R*^2^) and lower standard deviations (*SD*) values (Table 2), the pseudo-second-order model best described the adsorption behavior, with calculated capacities closely matching experimental data. The Avrami model also fitted well for KNBC, suggesting a mixed mechanism involving both physical and chemical adsorption [43]. Additionally, Elovich modeling yielded *α* > *β*, indicating that adsorption proceeded more rapidly than desorption. The intra-particle diffusion model revealed a three-stage uptake process, with curves deviating from the origin (inset of Figure 5d), implying multiple rate-limiting steps. Initially, GSM rapidly diffused across the biochar’s outer boundary, followed by gradual diffusion into internal pores and eventual equilibrium as adsorption reached inner surfaces [44]. The process was governed by a combination of external film diffusion and intra-particle diffusion [45]. The diffusion rate constants (*k_d_*) and intercepts (C) followed the order *k_d_*_1_ > *k_d_*_2_ > *k_d_*_3_ and C_1_ < C_2_ < C_3_, indicating that boundary-layer diffusion may be the primary rate-limiting step [46]. Notably, KOH activation enhanced *k_d_*_1_ and *k_d_*_2_, likely due to increased specific surface area and mesopore volume, which improved mass transfer and provided more active adsorption sites.

#### 3.3.4. Adsorption Isotherms

Four isotherm models were applied to fit the experimental data (Text S3, Equations (S8)–(S11), Figure 5e). The Langmuir and Freundlich models describe monolayer and multilayer adsorption on homogeneous and heterogeneous surfaces, respectively, while the Sips model accounts for heterogeneous adsorption behavior [47], and the Temkin model reflects interactions between adsorbate and adsorbent during adsorption dispersion [48]. Based on *R*^2^, both the Langmuir (0.992 for NBC and 0.98 for KNBC) and Sips (0.999 for NBC and 0.991 for KNBC) models provided good fits, indicating that GSM adsorption occurs primarily via monolayer adsorption on a heterogeneous surface (Table S8). This aligns with previous findings using activated spirulina carbon [15]. The dimensionless separation factor (*R_L_*) from the Langmuir model ranged between zero and one for all samples, confirming favorable adsorption [49], with *R_L_* values and Freundlich exponent (*n_F_*: 2.537 for NBC and 2.558 for KNBC) suggesting stronger adsorption affinity for KNBC over NBC (Figure S3). Additionally, the Langmuir constant (*K_L_*) of KNBC (0.345 L/μg) exceeded that of NBC (0.233 L/μg), further supporting its enhanced adsorption capacity. This improved performance is attributed to KNBC’s higher surface area, mesoporosity, and abundant functional groups. The exceptionally high Temkin binding energies (*b_T_* = 3255.64 and 3159.93 kJ/mol) suggest that GSM adsorption was largely governed by chemical interactions. Notably, the maximum adsorption capacity of KNBC reached 3.860 μg/g, significantly surpassing that of untreated moss biochar (58.41 ng/g) [14].

#### 3.3.5. Adsorption Thermodynamics

Thermodynamic parameters, such as enthalpy (Δ*H*^0^), Gibbs free energy (Δ*G*^0^), and entropy (Δ*S*^0^), were calculated to assess the energetics and spontaneity of GSM adsorption on NBCs (Text S3) [43]. As shown in Figure 5f and Table S9, the positive Δ*H*^0^ values indicate that the adsorption process was endothermic, consistent with isotherm results. Negative Δ*G*^0^ values confirm that GSM adsorption was spontaneous, while positive Δ*S*^0^ values suggest increased system randomness during adsorption.

Adsorption capacity increased with temperature (15–45 °C), a trend also observed in other systems such as GSM adsorption on granular activated carbon [50]. This endothermic behavior, contrary to typical exothermic physisorption, implies a chemisorption-dominated mechanism. Unlike physisorption, chemisorption involves surface bond formation, which can be enhanced at elevated temperatures due to bond breaking and the creation of new active sites. Additionally, increased molecular planarity of GSM at higher temperatures may facilitate its diffusion into the biochar pores, further promoting adsorption.

### 3.4. Regeneration and Practical Application

The regeneration ability of adsorbents is crucial for evaluating their practical and economic viability. In this study, GSM was desorbed from NBCs using methanol elution for 12 h. As a polar solvent, methanol weakens the adsorption energy of GSM via hydrogen bonding [51]. As shown in Figure 5g, after four regeneration cycles, the GSM adsorption capacity of KNBC decreased slightly from 3.81 μg/g (95.81%) to 3.54 μg/g (89.18%), and NBC from 3.74 μg/g (94.08%) to 3.43 μg/g (85.53%). The decline in adsorption was attributed to reductions in surface area, pore volume, and N-containing functional groups post-regeneration [52]. Notably, KNBC retained higher performance, highlighting its regeneration stability and potential for reuse in GSM removal.

To assess the practical applicability, adsorption tests were conducted using GSM-contaminated lake and aquaculture water. Compared with lake water, aquaculture water contains higher levels of nutrients and interfering substances (Figure S4) [53]. As shown in Figure 5h,i and Table 2, two adsorbents maintained good adsorption in both matrices. In lake water, KNBC and NBC achieved *q_e_* of 3.63 μg/g and 2.868 μg/g, respectively. In more complex aquaculture water, adsorption capacities slightly declined to 3.259 μg/g (KNBC) and 2.423 μg/g (NBC). The reduced performance is likely due to competition from coexisting organic and inorganic compounds for active sites. Nonetheless, KNBC demonstrated better resistance to interference, suggesting that KOH activation enhances selectivity and mass transfer via increased mesoporosity.

While powdered activated carbon remains widely used for removing odorous compounds like GSM and 2-MIB (2-methylisoborneol) in drinking water treatment, its application in aquaculture settings is limited. Table S10 compares the performance of various GSM adsorbents. Powdered activated carbon (PAC) remains the predominant adsorbent for GSM removal, with reported capacities ranging from 15 to 925.2 μg/g. However, its low mass transfer rate, dependence on physical pore filling, complex activation process, and high production cost limit its efficiency, particularly in urgent treatment situations. In contrast, the NBCs developed in this study, synthesized via hydrothermal carbonization of waste moss, offered a low-cost, sustainable alternative. Moss can be derived from harmful blue-green algae produced by eutrophication of pond water surfaces; the nitrogen raw material is cheap, and the doping and synthesis process is simple, with conditions for mass production. KNBC, in particular, demonstrated superior adsorption performance under high-impurity conditions, highlighting its strong potential for practical application in aquaculture water treatment, as well as in source water and landscape lake remediation.

### 3.5. Adsorption Mechanism

#### 3.5.1. Role of Pore Structure

**Figure 6:**
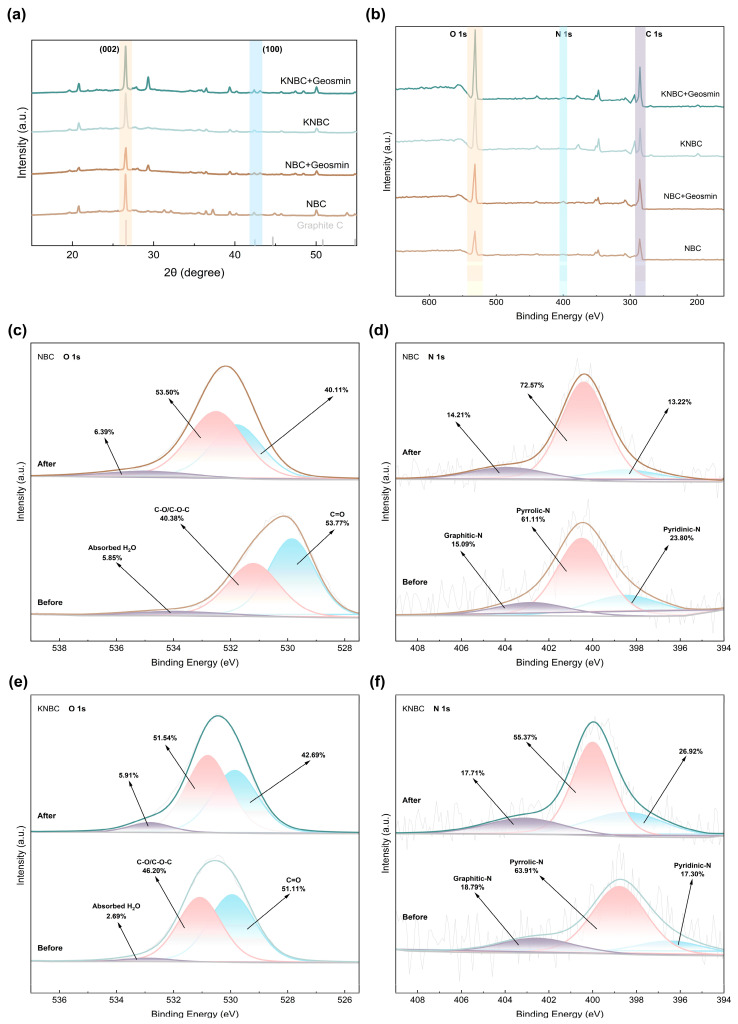
(**a**) The XRD and (**b**) XPS spectra of NBCs after adsorption of geosmin. (**c**) O 1s of NBC, (**d**) N 1s of NBC, (**e**) O 1s of KNBC, and (**f**) N 1s of KNBC.

XRD analysis (Figure 6a) showed characteristic diffraction peaks at 2θ = 26° and 42°, corresponding to graphitized carbon, which were retained after adsorption. The smoothing of the 42° peak post-adsorption suggests GSM binding to graphitic regions through π–π interactions and hydrophobic forces [36]. The absence of major crystalline phase changes confirms that the adsorption process did not significantly alter the carbon structure.

KOH activation significantly enhanced the adsorption capacity of NBCs by increasing their SSA, mesopore volume, and average pore size, indicating that mesopores play a dominant role in the pore diffusion mechanism. These structural features facilitate the rapid transport and uptake of GSM molecules.

#### 3.5.2. Role of Surface Functional Groups

XPS full-spectrum analysis (Figure 6b) confirmed the presence of C 1s, O 1s, and N 1s. After adsorption, increased signal intensities of C 1s and O 1s indicated efficient GSM capture. High-resolution O 1s spectra (Figure 6c,e) showed that binding energies of C=O and -OH shifted toward higher values, implying hydrogen bonding between the hydroxyl groups of GSM and the O-containing groups on the biochar (O-H⋯O=C) [54]. The stronger shift in NBC (~1 eV) compared to KNBC (~0.3 eV) suggests more pronounced chemical transformations during adsorption. Partial oxidation of -OH to -COOH was also observed. These findings confirm that O-containing functional groups serve as key adsorption sites through hydrogen bonding, particularly in NBC.

N-doping significantly improved GSM adsorption performance compared to pristine biochar (Table S10). Post-adsorption XPS spectra of N 1s (Figure 6d,f) revealed binding energy shifts (~0.8 eV), suggesting involvement of N-functional groups in electrostatic attraction, hydrogen bonding, and π–π donor–acceptor interactions [55]. Specifically, pyridinic-N may form weak hydrogen bonds with GSM, while graphitic-N, being electron-rich, facilitates π–π stacking with GSM’s bicyclic structure. These interactions are enhanced in KNBC due to its higher pyridinic-N and graphitic-N content (82.7%), compared to NBC (76.2%) [56].

**Figure 7:**
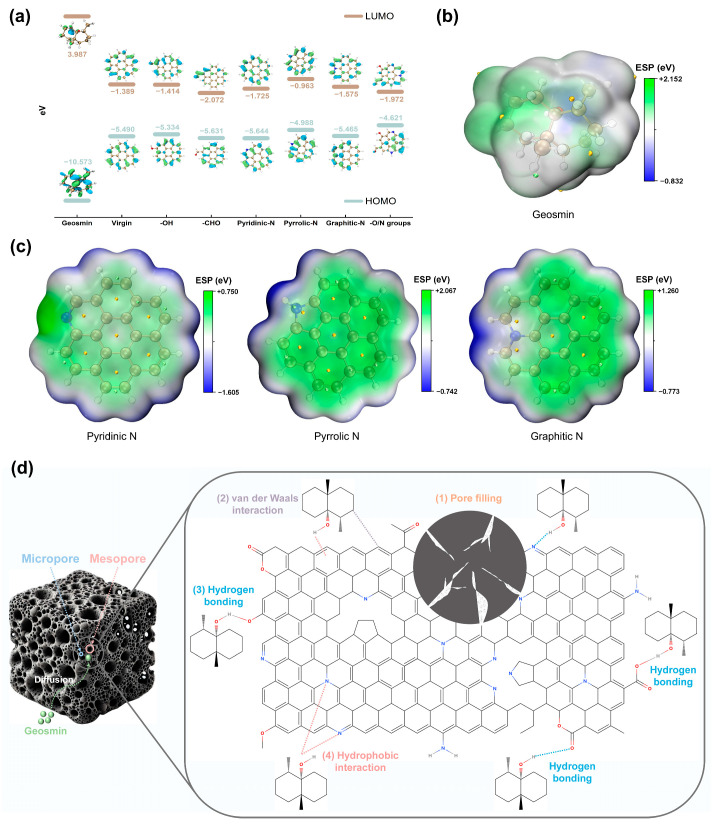
(**a**) Schematic diagram of Highest Occupied Molecular Orbital (HOMO) and Lowest Unoccupied Molecular Orbital (LUMO) for geosmin, virgin biochar, and modified biochar with O-/N-functional groups. Electron density distributions of (**b**) geosmin, (**c**) pyridinic N, pyrrolic N, and graphitic N. (**d**) Plausible adsorption mechanism of geosmin on KNBC.

By examining the frontier molecular orbitals (Highest Occupied Molecular Orbital: HOMO/Lowest Unoccupied Molecular Orbital: LUMO) of O-/N-containing functional groups in N-doped biochar and the adsorbate GSM, this study elucidates the dominant adsorption forces and their synergistic mechanisms from an electronic structure perspective. As indicated by Figure 7a, the HOMO–LUMO energy gap of N-doped biochar with O/N functional groups (2.649 eV) was significantly reduced compared to virgin biochar (4.101 eV), demonstrating enhanced electron excitation activity, improved conductivity, and heightened chemical reactivity induced by N-doping. The multifunctional structure exhibited triple synergistic effects: (1) strengthened conjugation effects facilitated delocalized electron cloud formation, (2) increased defect site density optimized charge transfer pathways, and (3) minimized bandgap (2.649 eV) lowered electron transition barriers. Although the energy gap between the LUMO of this system and the HOMO of GSM reached 8.601 eV, far exceeding the electron transfer threshold for chemisorption (<2 eV), the multifunctional groups synergistically enhanced physisorption by providing diverse binding sites, including hydrogen bonding networks, π-π stacking, and hydrophobic interactions [37]. Notably, the strong electron-withdrawing nature of -CHO (bandgap 3.559 eV) weakened hydrophobic adsorption but amplified dipole–dipole interactions. Similarly, pyrrole-N (bandgap 4.025 eV), constrained by its five-membered ring structure in π-electron delocalization, emerged as a critical adsorption site due to its superior hydrogen-bonding capability, which dominated GSM adsorption enhancement in N-doped hydrochar [57]. Electrostatic potential (ESP), a critical descriptor of electron density distribution and donor–acceptor tendencies [58], was simulated via DFT to evaluate GSM adsorption on N-doped graphene. As shown in Figure 7b,c, N-doping significantly altered the ESP distribution, with pyrrolic-N exhibiting the highest potential, followed by graphitic-N and pyridinic-N. Pyrrolic-N, capable of forming weak hydrogen bonds, was identified as the primary active site for GSM adsorption.

#### 3.5.3. Role of KOH Activation

The enhanced adsorption performance of KNBC is attributed to the synergistic effect of mesopore development and nitrogen functionalization. KOH activation created a more open porous structure, improving GSM diffusion and access to active sites. Simultaneously, it increased the content of pyrrolic-N and graphitic-N, which enhanced electrostatic attraction and π–π interactions.

In summary, GSM adsorption on NBCs involves multiple interactions, including hydrophobic effects, π–π stacking, van der Waals interactions, hydrogen bonding, and electrostatic forces. The primary adsorption mechanism was hydrophobic interaction, followed by hydrogen bonds and electrostatic interactions. In NBC, O-functional groups dominate through hydrogen bonding, with N-functional groups playing an auxiliary role. In contrast, KNBC relies primarily on N-doped active sites, especially pyrrolic-N, with O-functional groups serving as secondary contributors. These mechanisms collectively explain the superior adsorption performance of KNBC (Figure 7d).

## 4. Conclusions

N-doped hierarchical biochar with high SSA was successfully synthesized via AHT combined with KOH activation. RSM identified KNBC as the optimal material for GSM removal. The adsorption capacity was increased from 58.41 ng/g to 3933 ng/g. To elucidate the role of KOH activation, a non-activated counterpart (NBC) was prepared under identical conditions. AHT effectively introduced N functionalities and enhanced the structural rigidity of moss, improving its suitability as a biochar precursor. Compared to NBC, KNBC exhibited higher N-doping efficiency, increased SSA, and significantly improved GSM adsorption performance. The adsorption of geosmin in pond water by KNBC was 1.35 times higher than that of NBC. Adsorption behavior followed pseudo-second-order kinetics and conformed to the Langmuir isotherm model. DFT simulations and XPS analysis confirmed that N-/O-containing functional groups, particularly pyrrolic-N and graphitic-N, served as key adsorption sites. Overall, KNBC offers a promising and sustainable approach for odorant control in water treatment while also valorizing aquaculture-derived biomass waste.

## Figures and Tables

**Table 1:** Textural parameters of biochar.

Biochar	BET Surface Area (m^2^/g) ^a^	Langmuir Surface Area (m^2^/g)	Average Pore Diameter (nm)	Pore Volume (cm^3^/g)	Mesopore Area (m^2^/g)	Mesopore Diameter (nm) ^a^	Mesopore Volume (cm^3^/g) ^a^	Micropore Area (m^2^/g)	Micropore Volume (cm^3^/g) ^a^	Mesopore Volume/Pore Volume (%)
BC	18.55	/	9.584	0.044	8.954	4.302	0.040	2.394	0.000	90.91
NBC	57.05	148.10	5.655	0.081	21.489	12.139	0.065	32.441	0.014	80.25
KNBC	62.13	267.33	14.029	0.218	37.179	22.203	0.206	22.354	0.010	94.50

^a^ The specific surface area, the mesopore volume, and the micropore volume were determined by the methods of the Brunauer–Emmett–Teller (BET) method, the Barrett–Joyner–Halenda (BJH) method, and the t-plot method, respectively.

**Table 2:** Kinetic model parameters for the adsorption of geosmin onto NBC and KNBC in various water bodies.

Water Bodies	Biochar	*q_e,exp_* (μg/g)	Pseudo-First-Order				Pseudo-Second-Order				Avrami Fractional Order					Elovich			
			*q_e,cal_* (μg/g)	*k*_1_ (min^−1^)	*R* ^2^	*SD*	*q_e,cal_* (μg/g)	*k*_2_ (g/μg·min^−1^)	*R* ^2^	*SD*	*q_e,cal_* (μg/g)	*k*_3_	*n*	*R* ^2^	*SD*	*α* (μg/g·min^−1^)	*β* (μg/g)	*R* ^2^	*SD*
Purified water	NBC	3.807	3.679	6.504	0.996	0.071	3.707	2.288	0.997	0.059	4.919	0.250	0.114	0.999	1.184	2.778 × 10^5^	4.989	0.999	0.316
	KNBC	3.933	3.910	2.172	0.999	0.020	3.927	0.905	0.999	0.014	3.952	4.779	0.259	0.999	0.006	1.480 × 10^16^	11.311	0.999	2.055
Lake water	NBC	2.868	2.639	3.106	0.978	0.118	2.711	1.669	0.989	0.089	3.053	0.767	0.214	0.999	0.111	2.153 × 10^3^	4.966	0.998	0.235
	KNBC	3.630	3.359	1.706	0.972	0.168	3.442	0.672	0.983	0.139	5.491	0.011	0.173	0.999	1.856	221.098	3.203	0.998	0.147
Aquaculture water	NBC	2.423	2.179	2.418	0.988	0.075	2.240	1.922	0.994	0.054	2.508	1.488	0.222	0.999	0.073	3.764 × 10^3^	5.987	0.999	0.308
	KNBC	3.259	3.155	0.495	0.987	0.110	3.222	0.244	0.992	0.090	3.756	0.129	0.232	0.999	0.399	127.795	3.429	0.998	0.174

Note: *q_e,exp_*—*q_e,experimental_*; *q_e,cal_*—*q_e,calculated_*.

## Data Availability

The data presented in this study are available on request from the corresponding authors.

## Supplementary Materials

The following supporting information can be downloaded at: https://www.mdpi.com/article/10.3390/biology14081045/s1, Text S1. Characterization of biochar; Text S2. Geosmin analysis in water; Text S3. Adsorption models of geosmin; Text S4. Density functional theory (DFT) calculations; Figure S1. Effects of (a) mass ratio and (b) hydrothermal temperature on the adsorption of geosmin by KNBCs; Figure S2. Effect of NBCs’ dosage on geosmin adsorption; Figure S3. Dimensionless constant (*R_L_*) of geosmin adsorbed onto NBCs; Figure S4. Water samples from lake (Li Lake, located in Wuxi, Jiangsu Province) and aquaculture pond (fish farming pond in Wuxi, Jiangsu Province); Figure S5. The XPS spectra of NBCs after adsorption of geosmin. (a) C 1s of NBC, (b) C 1s of KNBC; Figure S6. The SEM full size versions of BCs; Table S1. Determination of factor levels; Table S2. Experimental factors and levels of phenol adsorption by adsorbents; Table S3. Experimental design matrix and experiment-based response values; Table S4. ANOVA table for KNBCs response to geosmin adsorption; Table S5. Statistics of geosmin adsorption by KNBCs; Table S6. Optimization solutions for experimental conditions; Table S7. Element content distribution of biochar; Table S8. Isotherm parameters for geosmin adsorption onto NBCs at 298.15 K; Table S9. Thermodynamics parameters for the uptake of geosmin on NBCs; Table S10. Comparison of the removal efficiencies of geosmin using carbon materials in real water sources; Table S11. Physical–chemical properties of geosmin [59,60,61,62,63,64,65,66].

## Abbreviations

The following abbreviations are used in this manuscript:

BC	Unmodified moss biochar
NBC	N-doped moss biochar
KNBC	KOH-activated N-doped moss biochar
