# Supplementary material for: N-Doping/KOH Synergy in Waste Moss Biochar for Geosmin Removal in Aquaculture Water: Elucidating Surface Functionalization and Activation Mechanisms
Source: Biology (Basel). 2025 Aug 14;14(8):1045. doi: 10.3390/biology14081045 (PMC12383663; doi:10.3390/biology14081045)
Supplement: Supplementary file 1 [file biology-14-01045-s001.zip › biology-3805158-supplementary.pdf]

Supplementary Information

N-doping/KOH synergy in aquaculture moss biochar for geosmin adsorption: Elucidating surface functionalization and activation mechanisms

Zhonghua Li[1], Xi Chen[2], Liping Qiu[2,3,4], Huimin Xu[2,3,4], Limin Fan[1,2,3,4], Shunlong Meng[1,2,3,4], Zhongquan Jiang[5,6] Chao Song[1,2,3,4*]

[1] Wuxi Fisheries College, Nanjing Agricultural University, 214081 Wuxi, China

[2] Freshwater Fisheries Research Center, Chinese Academy of Fishery Sciences, 214081 Wuxi, China

[3] Laboratory of Quality & Safety Risk Assessment for Aquatic Products on Environmental Factors (Wuxi), Ministry of Agriculture and Rural Affairs, 214081 Wuxi, China

[4] Key Laboratory of Control of Quality and Safety for Aquatic Products, Ministry of Agriculture and Rural Affairs, 100141 Beijing, China

[5] Key Laboratory of Environmental Health Impact Assessment of Emerging Contaminants, Ministry of Ecology and Environment, School of Environmental Science and Engineering, Shanghai Jiao Tong University, Shanghai 200240, China

[6] East China Sea Fisheries Research Institute, Chinese Academy of Fishery Sciences, Shanghai 200090, China

[*] Corresponding author: Prof. C Song (songc@ffrc.cn)

Content

Texts:

Text S1. Characterization of biochar

Text S2. Geosmin analysis in water

2.1 Sample pretreatment

2.2 Instrumental analysis

2.3 Quality assurance and quality control

Text S3. Adsorption models of geosmin

3.1 Adsorption kinetics

3.2 Adsorption isotherms

3.3 Adsorption thermodynamics

Text S4. Density functional theory (DFT) Calculations.

Figures:

Figure S1. Effects of (a) mass ratio and (b) hydrothermal temperature on the adsorption of geosmin by KNBCs

Figure S2. Effect of NBCs dosage on geosmin adsorption

Figure S3. Dimensionless constant (R_L) of geosmin adsorbed onto NBCs

Figure S4. Water samples from lake (Li Lake, located in Wuxi, Jiangsu Province) and aquaculture pond (fish farming pond, in Wuxi, Jiangsu Province)

Figure S5. The XPS spectra of NBCs after adsorption of geosmin. (a) C 1s of NBC, (b) C 1s of KNBC

Tables:

Table S1. Determination of factor levels

Table S2. Experimental factors and levels of phenol adsorption by adsorbents

Table S3. Experimental Design Matrix and Experiment-Based Response Values

Table S4. ANOVA table for KNBCs response to geosmin adsorption

Table S5. Statistics of geosmin adsorption by KNBCs

Table S6. Optimization solutions for experimental conditions

Table S7. Element content distribution of biochar

Table S8. Isotherm parameters for geosmin adsorption onto NBCs at 298.15 K
Table S9. Thermodynamics parameters for the uptake of geosmin on NBCs
Table S10. Comparison of the removal efficiencies of geosmin using carbon materials in real water sources
Table S11. Physical-chemical properties of geosmin

Text S1. Characterization of biochar

The thermal stability of the materials was analyzed using a thermogravimetric analyzer (STA 449 F5, Netzsch, Germany). The ultimate analysis of the sample was determined using an elemental analyzer (Unicube, Elementar, Germany). The surface microstructure of sample was analyzed using the scanning electron microscope (GeminiSEM 300, ZEISS, Germany). The Brunauer–Emmett–Teller (BET) surface area, pore size, and pore volume of the sample were analyzed with an Automatic Surface Area and Pore Analyzer (ASAP 2460, Micromeritics, USA). The biochars structure was characterized by X-ray Diffraction (Ultima IV, Rigaku, Japan) with Cu Kα radiation, scanning was run from 5° to 90° (2θ). The surface functional group of the sample was analyzed with an FT-IR spectrophotometer (Nicolet iS20, Thermo Fisher Scientific, USA). The surface chemical states of the catalyst were analyzed by X-ray photoelectron spectrometry (XPS) using a K-Alpha spectrometer with Al Kα radiation (Nexsa, Thermo Fisher Scientific, USA).

Text S2. Geosmin analysis in water

2.1 Sample pretreatment

We employed headspace solid phase microextraction (HS-SPME) in conjunction with gas chromatography-mass spectrometry (GC-MS) to detect odor substances in the water samples. Specifically, a ratio of 4:1 (water to salt) was achieved by introducing 2 g of sodium chloride to a 10 mL sample of water housed in a 15 mL vial with solid-phase headspace. After placing the mixture on a magnetic stirrer, set at 1200 r/min, and kept at a constant temperature of 60 °C, the solution was allowed to extract for 30 minutes. Subsequently, the extraction fiber head was injected into the injection port of the GC-MS for further analysis.

2.2 Instrumental analysis

The chromatographic and mass spectrometric conditions of the GC-MS were as follows: DB-5 column (30 m×0.25 mm×0.25 μm), with the following temperature-rise procedure: the starting temperature was 50 °C, held for 1 min, then increased to 120 °C at the rate of 10 °C/min, held for 1 min, and then increased to 220 °C at the rate of 20 °C/min, held for 1 min; the injection mode was non-split injection; the temperature of the inlet port was 250 °C; the pressure of the inlet port was 7.66 psi; the total flow rate was 44 mL/min; the carrier gas was high purity helium (purity > 99.999%) at a flow rate of 1.0 mL/min. The electron ionization source was used with an ionization energy of 70 eV; the ionization temperature was 230 °C; the temperature of the MS quadrupole was 150 °C; the temperature of the transfer line was 250 °C; and the solvent was delayed for 5 min. The scanning mode was selected as ion detection (SIM).

2.3 Quality assurance and quality control

The limits of detection (LODs) and limits of quantification (LOQs) were defined as the concentrations corresponding to a signal-to-noise ratio of 3 and 10. The LODs and LOQs of geosmin in water were 0.5-1.0 ng/L and 1.5-2.0 ng/L, respectively.

Recoveries of geosmin were 88.4-109.7% in the water samples. Precision was assessed using the relative standard deviation (RSD) of six replicate treatments. The methodology was generally well reproducible, with RSDs ranging from 1.7 to 14.2%.

Text S3. Adsorption models of geosmin

3.1 Adsorption kinetics

The removal rate (R_r) and adsorption capacity were calculated as follows:

$$R_r = \frac{(C_0\text{-}C_e)}{C_0} \times 100\% \quad \text{(S1)}$$

Adsorption capacity at moment t:

$$q_t = \frac{(C_0\text{-}C_t)}{m} \times V \quad \text{(S2)}$$

Where q_e (μg/g) and q_t (μg/g)) are the number of pollutants adsorbed by the biochars at equilibrium and at time t, respectively; C_0 (μg/L) and C_e (μg/L) represent the initial and equilibrium concentrations of pollutants, respectively.

The equations and associated parameters for the three dynamics are given below:

Pseudo-first-order:

$$q_t = q_e(1 - e^{-k_1 t}) \quad \text{(S3)}$$

Pseudo-second-order:

$$q_t = \frac{t k_2 q_e^2}{1 + t q_e k_2} \quad \text{(S4)}$$

Avrami fractional-order:

$$q_t = q_e\left[1 - e^{-(k_3 t)^n}\right] \quad \text{(S5)}$$

Elovich:

$$q_t = \frac{1}{\beta}\ln(\alpha\beta t + 1) \quad \text{(S6)}$$

Intra-particle diffusion:

$$q_t = k_i t^{0.5} + C_i \quad \text{(S7)}$$

The variables in question are as follows: t is the adsorption time, min; q_e denotes the equilibrium adsorption amount, μg/g; q_t denotes the adsorption amount at time t, μg/g; k_1 is the proposed first-order kinetic coefficient, min^{-1}; k_2 denotes the proposed second order adsorption rate constant, g/μg·min $^{-1}$; k_3 denotes the constant, and n is the Avrami index, generally in the range 1 to 4; α (mol/g·min) and β as the elovich constants represent the initial adsorption rate and the desorption coefficient; k_i and C_i are the pore rate constant (g/μg·$min^{-0.5}$) and the constant, respectively.

3.2 Adsorption isotherms

To further investigate the adsorption pattern and adsorption mechanism of antibiotics on different biochars, adsorption isothermal equations were fitted with the Langmuir and Freundlich models, respectively.

Langmuir:

$$q_e = \frac{K_L q_{max} C_e}{1 + K_L C_e} \tag{S8}$$

Freundlich:

$$q_e = K_F C_e^{\frac{1}{n_F}} \tag{S9}$$

Sips:

$$q_e = \frac{K_S C_e^{n_S}}{1 + a_S C_e^{n_S}} \tag{S10}$$

Temkin:

$$q_e = \frac{\mathrm{RT}}{b_T} \ln(K_T C_e) \tag{S11}$$

where, the equilibrium and saturation quantity of geosmin captured by biochars are represented by q_e and q_{max}, K_L, K_F, and K_S are correlated with the coefficients of models, n_F and n_S mean corresponding indexes, K_T is the equilibrium binding constant, b_T is Temkin constant related to heat of adsorption, R is the gas constant (8.314 J/mol·K), C_e are the concentration of the un-adsorbed geosmin at equilibrium, while T is the absolute temperature.

3.3 Adsorption thermodynamics

The adsorption thermodynamic parameters were investigated at temperatures of 288.15 K, 298.15 K, 308.15 K, and 318.15 K. The changes in Gibbs free energy (ΔG^0), enthalpy (ΔH^0), and entropy (ΔS^0), which are related to the feasibility and nature of the adsorption process, are expressed using the following equations.

$$\Delta G^0 = -\mathrm{RT}\ln(K_0) = \Delta H^0 - T\Delta S^0 \tag{S12}$$

$$\ln K_0 = \frac{\Delta S^0}{R} - \frac{\Delta H^0}{RT} \tag{S13}$$

$$K_0 = \frac{q_e}{C_e} \tag{S14}$$

in which, K_0 as a thermodynamic equilibrium constant can be calculated by Langmuir isotherm; R is ideal gas constant, 8.314 J/(mol·K); q_e is the equilibrium adsorption amount (μg/g), and C_e is the equilibrium concentration (μg/L); T is thermodynamic temperature (K).

Text S4. Density functional theory (DFT) Calculations.

The molecular orbital and electrostatic potential analyses were done using density functional theory (DFT) by Gaussian 16. The B3LYP/6-31G(d) method was first used to optimize the geometry of the target molecule, and after confirming the absence of imaginary frequencies, single-point energy calculations were executed at the same level of theory to obtain the HOMO and LUMO energy levels and orbital distributions. Electrostatic potential (ESP) analysis was performed to visualize the potential distribution on the surface of the molecule using GaussView 6.0, and the maximum positive and maximum negative potential values were extracted from the output file. The spatial correlation between HOMO/LUMO and ESP was verified by superimposing the orbital isosurfaces with the potential isosurface plots. The ESP isosurface maps were visualized using Multiwfn and Visual Molecular Dynamics (VMD) software to show the potential distribution on the molecular surface (blue/green for negative/positive potentials, respectively) Energy level values were extracted from the output file and converted to eV units.

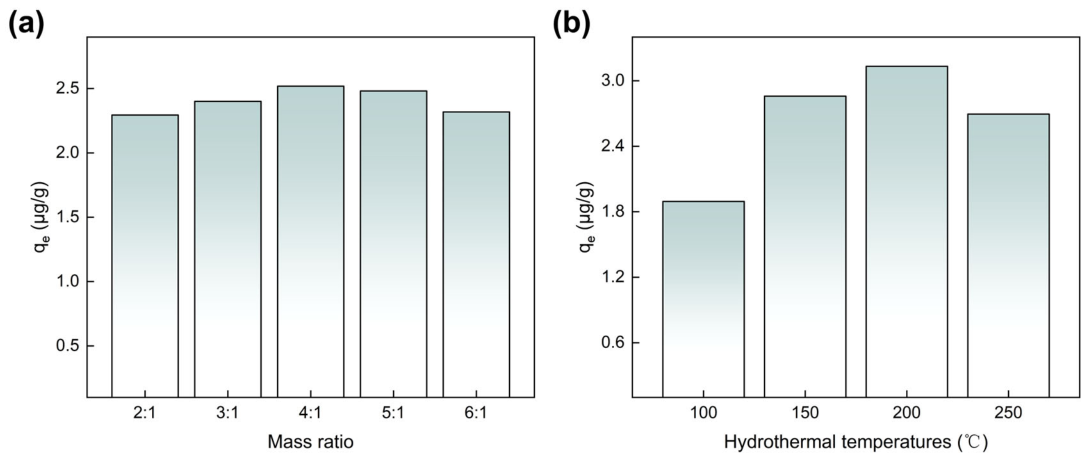

Figure S1. Effects of (a) mass ratio and (b) hydrothermal temperature on the adsorption of geosmin by KNBCs.

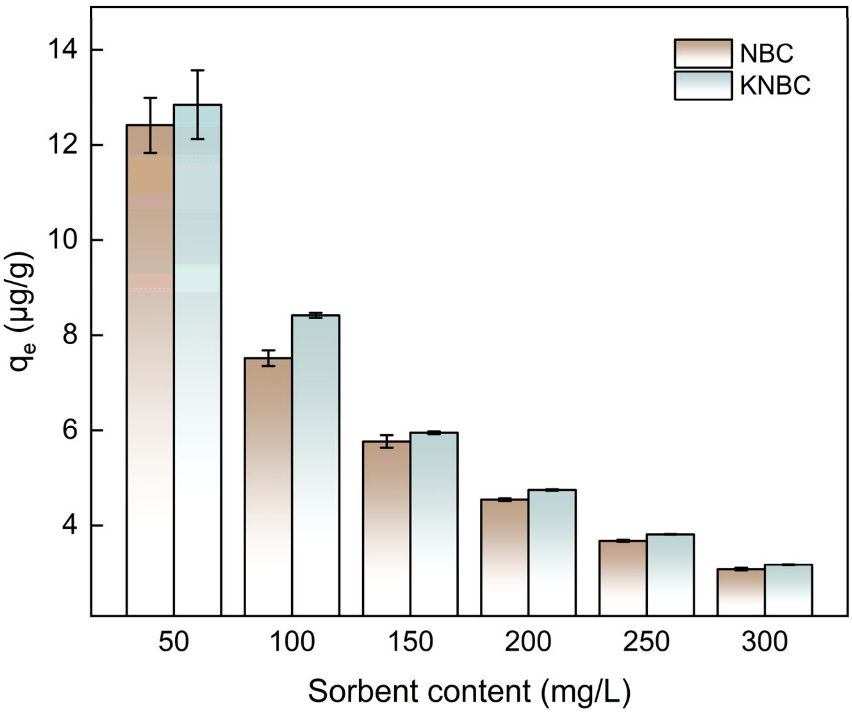

Figure S2. Effect of NBCs dosage on geosmin adsorption.

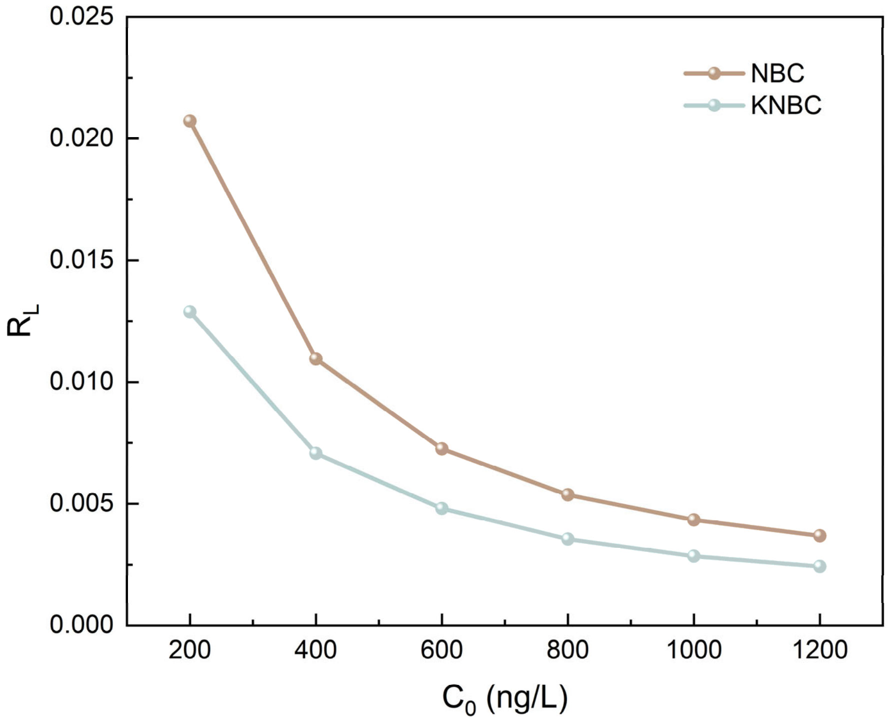

Figure S3. Dimensionless constant (R_L) of geosmin adsorbed onto NBCs.

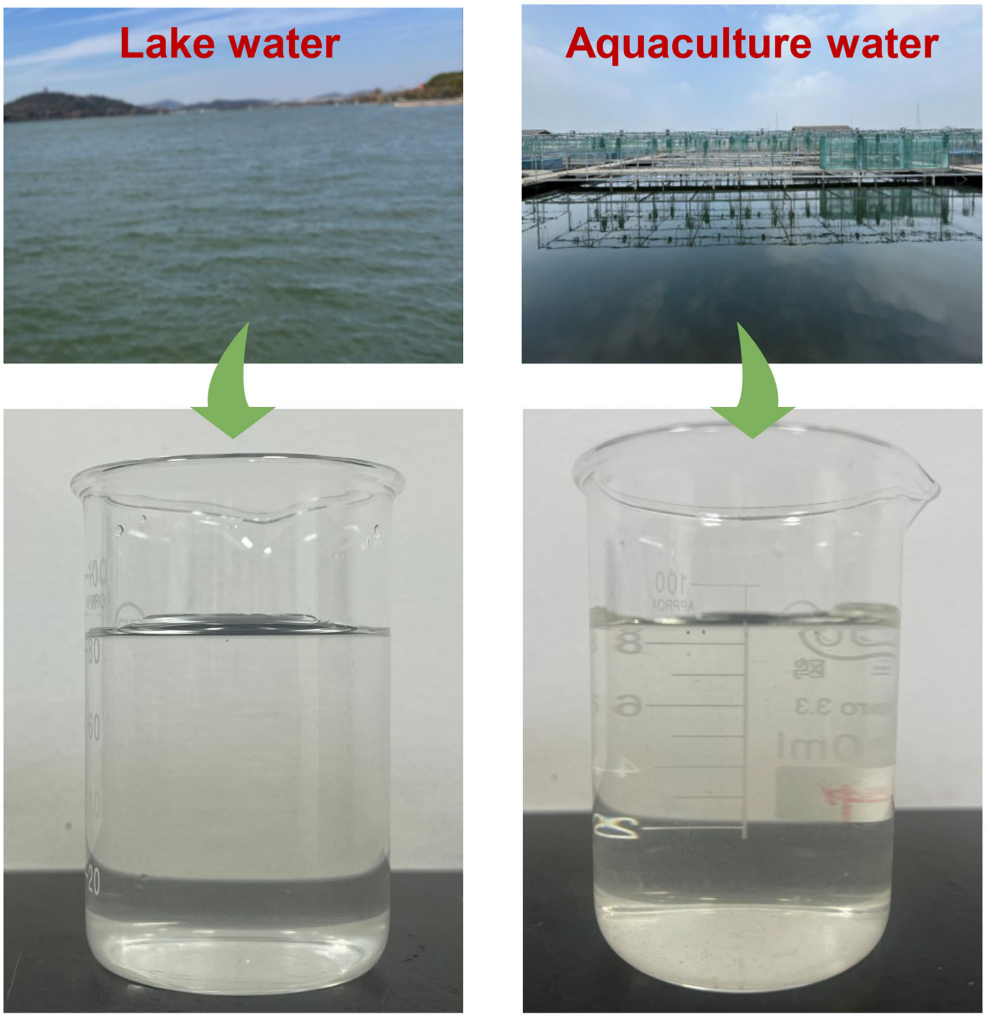

Figure S4. Water samples from lake (Li Lake, located in Wuxi, Jiangsu Province) and aquaculture pond (fish farming pond, in Wuxi, Jiangsu Province).

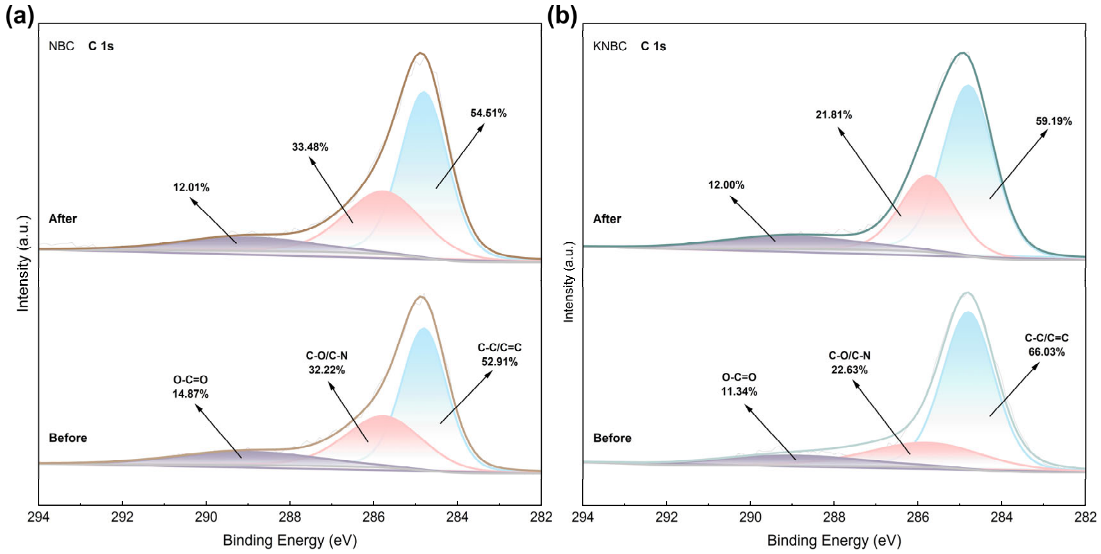

Figure S5. The XPS spectra of NBCs after adsorption of geosmin. (a) C 1s of NBC, (b) C 1s of KNBC.

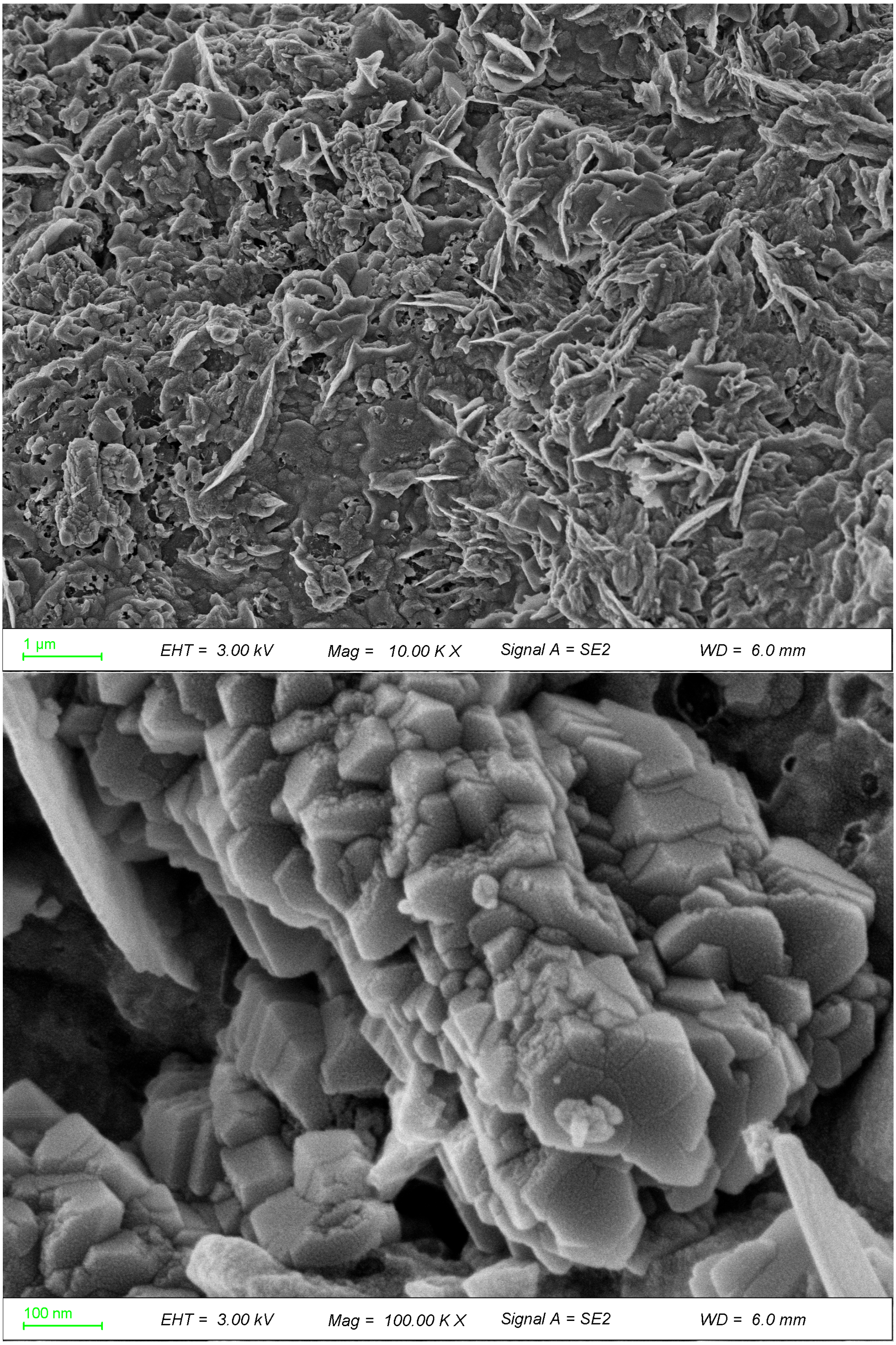

SEM image of BC

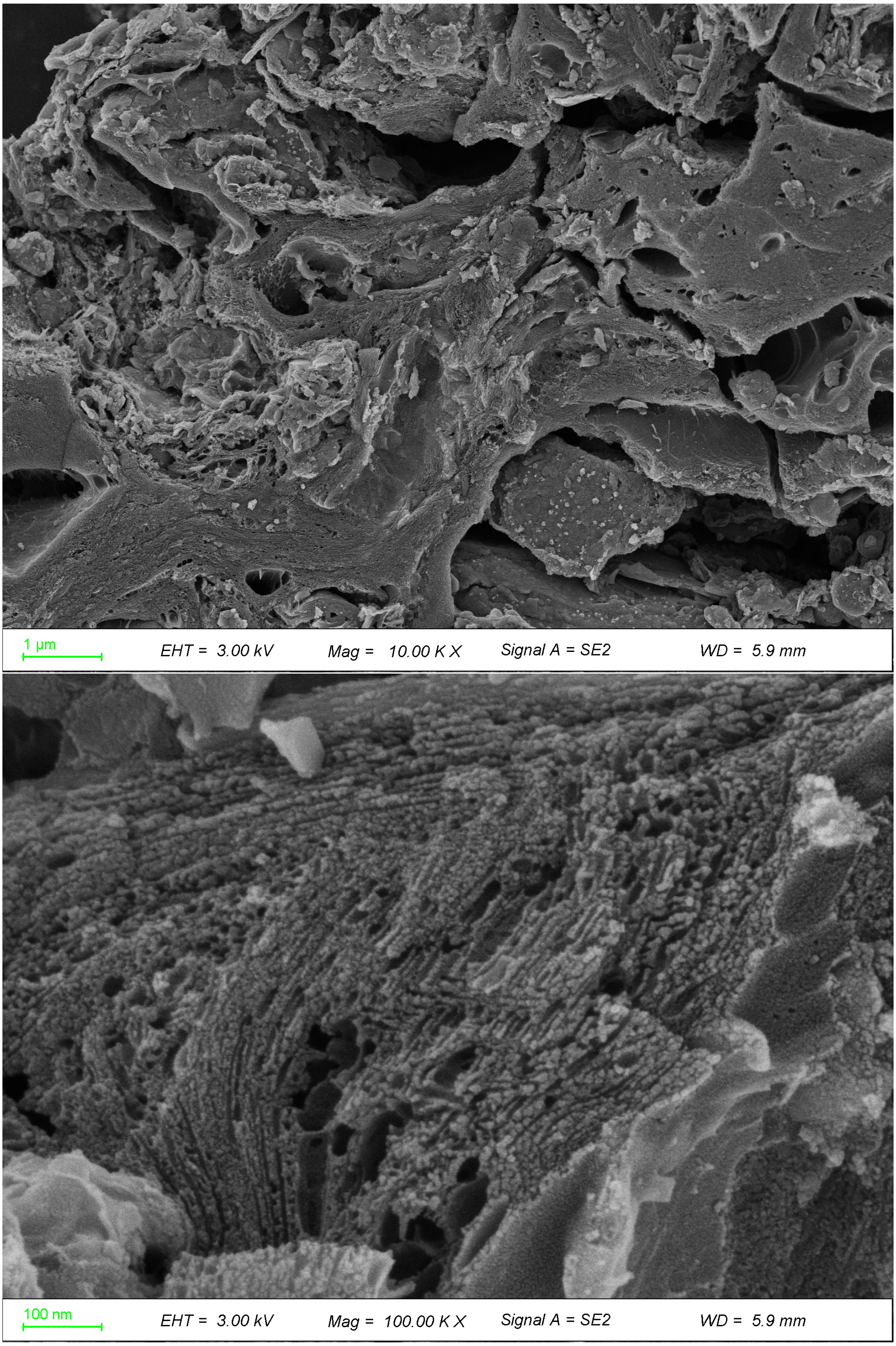

SEM image of NBC

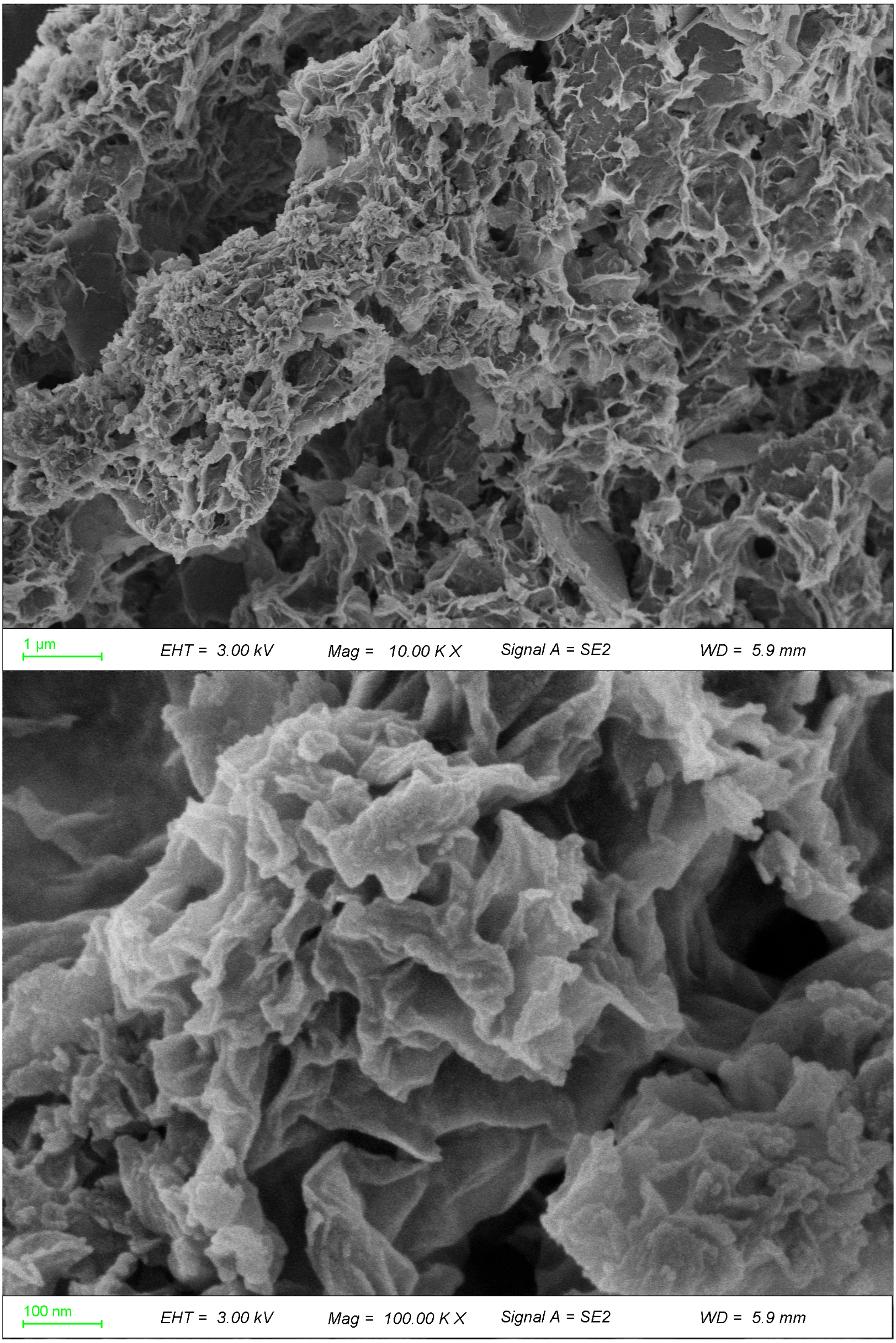

SEM image of KNBC

Figure S6. The SEM full size versions of BCs.

Table S1. Determination of factor levels.

Factors	Determination of factor levels
A-Biomass/ nitrogen	Hydrothermal treatment at 150 °C for 1 h, followed by pyrolysis at 800 °C (heating rate: 10 °C/min, holding time: 2 h). Biomass-to-nitrogen mass ratios (w/w) of 2:1, 3:1, 4:1, 5:1, and 6:1 was investigated in Figure S1.
B-Hydrothermal temperature	With a fixed biomass-to-nitrogen mass ratio of 4:1 and pyrolysis conditions set at 800 °C (heating rate: 10 °C/min, holding time: 2 h), hydrothermal temperatures of 100 °C, 150 °C, 200 °C, and 250 °C were studied in Figure S1.
C-Pyrolysis temperature	The pyrolysis temperatures of the moss were set at 400, 600 and 800°C according to the change rule of the quality of the moss in the range of 30-1000°C in figure 3a.

Table S2. Experimental factors and levels of phenol adsorption by adsorbents.

Independent variable	Coded	Unit	Factorial and center level		
			Low (-1)	Center	High (+1)
Biomass/ nitrogen	A	-	3	4	5
Hydrothermal temperature	B	℃	150	200	250
Pyrolysis temperature	C	℃	400	600	800

Table S3. Experimental Design Matrix and Experiment-Based Response Values.

Run	A: Biomass/ nitrogen -	B: Hydrothermal temperature ℃	C: Pyrolysis temperature ℃	q_e: Equilibrium adsorption capacity μg/g
1	4	150	800	3.829
2	3	250	600	2.39
3	4	200	600	2.656
4	4	250	400	1.484
5	5	150	600	2.808
6	4	200	600	2.649
7	4	200	600	2.651
8	3	200	800	3.214
9	4	250	800	2.856
10	5	250	600	2.473
11	5	200	800	3.479
12	4	200	600	2.511
13	3	200	400	1.31
14	5	200	400	1.428
15	3	150	600	2.494
16	4	200	600	2.639
17	4	150	400	1.244

Table S4. ANOVA table for KNBCs response to geosmin adsorption.

Source	Sum of Squares	df	Mean Square	F-value	p-value	
Model	8.70	9	0.9664	258.58	< 0.0001	**significant**
**A**	0.0761	1	0.0761	20.35	0.0028	
**B**	0.1717	1	0.1717	45.94	0.0003	
**C**	7.82	1	7.82	2093.65	< 0.0001	
AB	0.0133	1	0.0133	3.57	0.1008	
AC	0.0054	1	0.0054	1.45	0.2683	
**BC**	0.3678	1	0.3678	98.42	< 0.0001	
A^2	0.0060	1	0.0060	1.60	0.2459	
B^2	0.0075	1	0.0075	2.01	0.1993	
**C^2**	0.2145	1	0.2145	57.40	0.0001	
Residual	0.0262	7	0.0037			
Lack of Fit	0.0108	3	0.0036	0.9417	0.4996	**not significant**
Pure Error	0.0153	4	0.0038			
Cor Total	8.72	16				

Table S5. Statistics of geosmin adsorption by KNBCs.

Fit Statistics	Value
Std. Dev.	0.0611
Mean	2.48
C.V. %	2.47
R^2	0.9970
Adjusted R^2	0.9931
Predicted R^2	0.9774
Adeq Precision	55.1204

Note: The **Predicted R^2** of 0.9774 is in reasonable agreement with the **Adjusted R^2** of 0.9931; i.e. the difference is less than 0.2. **Adeq Precision** measures the signal to noise ratio. A ratio greater than 4 is desirable. The ratio of 55.120 indicates an adequate signal. This model can be used to navigate the design space.

Table S6. Optimization solutions for experimental conditions.

Number	A: Biomass/ nitrogen	B: Hydrothermal temperature ℃	C: Pyrolysis temperature ℃	q_e: Equilibrium adsorption capacity μg/g	Desirability	
**1**	**5.000**	**150.000**	**800.000**	**3.946 (calculated)**<br>**3.933 (experimental)**	**0.686**	**Selected**
2	4.993	150.000	800.000	3.945	0.681	
3	5.000	150.363	800.000	3.943	0.668	
4	4.957	150.000	800.000	3.941	0.656	
5	5.000	150.772	799.965	3.940	0.647	
6	4.940	150.000	800.000	3.939	0.644	
7	5.000	150.088	798.486	3.939	0.643	
8	4.950	150.233	800.000	3.938	0.640	
9	4.927	150.000	800.000	3.938	0.635	
10	4.919	150.000	800.000	3.937	0.629	
11	4.885	150.000	800.000	3.932	0.605	
12	5.000	151.688	800.000	3.932	0.602	
13	4.845	150.000	800.000	3.927	0.575	
14	5.000	152.104	799.557	3.926	0.570	
15	4.838	150.000	800.000	3.926	0.569	
16	4.825	150.000	800.000	3.925	0.560	
17	4.812	150.000	800.000	3.923	0.550	
18	5.000	152.857	800.000	3.922	0.544	
19	5.000	150.000	794.270	3.921	0.538	
20	4.748	150.000	800.000	3.915	0.500	
21	5.000	150.049	792.807	3.914	0.497	
22	5.000	153.900	800.000	3.913	0.491	
23	4.685	150.000	800.000	3.906	0.449	
24	5.000	150.000	790.781	3.905	0.446	
25	5.000	150.676	791.101	3.901	0.422	
26	5.000	150.000	788.474	3.895	0.386	
27	4.600	150.000	800.000	3.894	0.378	
28	4.589	150.000	800.000	3.892	0.368	
29	5.000	156.473	800.000	3.891	0.361	
30	4.574	150.000	800.000	3.890	0.355	
31	4.538	150.000	800.000	3.884	0.323	
32	5.000	157.384	799.999	3.883	0.315	
33	5.000	150.000	782.567	3.868	0.228	

34	5.000	159.952	800.000	3.860	0.184
35	4.442	150.000	797.235	3.858	0.167
36	4.261	150.000	800.000	3.839	0.061
37	5.000	156.795	787.222	3.834	0.027
38	4.224	150.000	800.000	3.833	0.024
39	4.215	150.000	800.000	3.832	0.015

Table S7. Element content distribution of biochar.

Biochar	Element composition (%)					Atomic ratio		
	C	N	O[a]	H	S	O/C	H/C	(N+O)/C
BC	36.71	1.53	8.46	1.49	4.34	0.23	0.041	0.27
NBC	7.94	0.37	6.81	0.22	1.52	0.86	0.028	0.90
KNBC	9.52	0.40	14.52	0.68	0.43	1.53	0.071	1.57

[a] Oxygen content was obtained using a differential method.

Table S8. Isotherm parameters for geosmin adsorption onto NBCs at 298.15 K.

Biochar	Langmuir			Freundlich			Sips				Temkin		
	q_{max} (μg/g)	K_L (L/μg)	R^2	K_F (μg/g) (L/μg)$^{1/n}$	n_F	R^2	K_S (L/μg)	a_S	n_S (J/mol K^{-1})	R^2	K_T (L/μg)	b_T (kJ/mol)	R^2
NBC	3.768	0.233	0.992	0.871	2.537	0.913	0.911	0.213	0.841	0.999	2.566	3255.64	0.996
KNBC	3.860	0.345	0.980	0.962	2.558	0.880	1.227	0.256	0.776	0.991	3.611	3159.93	0.983

Table S9. Thermodynamics parameters for the uptake of geosmin on NBCs.

Biochar	T (°C)	T (K)	LnK_0	ΔG^0 (KJ/mol)	ΔH^0 (KJ/mol)	ΔS^0 (J·mol^{-1}·K^{-1})
NBC	15	288.15	4	-9.57	8.38	62.19
	25	298.15	4.07	-10.10		
	35	308.15	4.24	-10.86		
	45	318.15	4.31	-11.39		
KNBC	15	288.15	4.39	-10.52	13.05	81.56
	25	298.15	4.52	-11.20		
	35	308.15	4.68	-12.00		
	45	318.15	4.91	-12.98		

Table S10. Comparison of the removal efficiencies of geosmin using carbon materials in real water sources.

Real water sources	Initial concentration of geosmin (ng/L)	Type of carbon materials	Dose of materials (mg/L)	Contact time	q_m (or removal efficiency) (μg/g)	References
Reservoir	100	C-PAC	10	3-5 days	93-95%	[1]
Natural water	100	S-PAC<br>W-PAC<br>Fruit shell-based PAC	2-30	3-5 days	24-26<br>37<br>58	[2]
Influent from WTP	100	S-PAC	30	Stirring for 40 min and settling for 30 min	95%	[3]
River water	100	C-PAC	32.14	20 min	> 80%	[4]
River water	200	WPH/WC	25	240 min	> 98%	[5]
Raw water	10^5	PAC	500	90 min	925.2 (94%)	[6]
Raw water	114	CS-PAC	2-8	30 min	15-25%	[7]
Purified water	300	BC800	5000	2 min	58.41ng/g (97.08%)	[8]
Raw water<br>Purified water	20	ASBCM800	10	240 min	64 %<br>2.353	[9]
Purified water with HA (20 ng/L)	200	NBC<br>KNBC	100	120 min	2.015 (91.57%)<br>2.214 (91.75%)	This study
Lake water	1000	NBC<br>KNBC	250		2.868 (71.17%)<br>3.630 (90.46%)	
Aquaculture water		NBC<br>KNBC			2.423 (60.19%)<br>3.259 (81.16%)	

Note: Biochar-BC/NBC/KNBC, Powdered activated carbon-PAC/WPH/WC.

Table S11. Physical-chemical properties of geosmin.

Parameters	Geosmin (GSM)	Symbol	Units
Molecular formula	$C_{12}H_{22}O$	/	/
CAS	16423-19-1	/	/
Molecular weight	182.3 [a]	M_r	g mol^{-1}
Melting point	47.08 [a]	*m.p.*	℃
Solubility	150.2 [b]	S	mg L^{-1}
Density	0.9494 [b]	ρ	g cm^{3}
Vapor pressure	5.56 [b]	P_s	Pa
Henry's constant	6.75 [b]	H	Pa m^{3} mol^{-1}
Logarithm of octanol-water partition coefficient	3.75 [c]	Log K_{ow}	/

[a] (Chemical-book, 2021); [b] [10]; [c] [11]; "/" represents no values.

References

1. Cook, D.; Newcombe, G.; Sztajnbok, P. The application of powdered activated carbon for mib and geosmin removal: predicting pac doses in four raw waters. *Water Research* **2001**, *35*, 1325-1333, doi:https://doi.org/10.1016/S0043-1354(00)00363-8.
2. Yu, J.; Yang, M.; Lin, T.-F.; Guo, Z.; Zhang, Y.; Gu, J.; Zhang, S. Effects of surface characteristics of activated carbon on the adsorption of 2-methylisobornel (MIB) and geosmin from natural water. *Separation and Purification Technology* **2007**, *56*, 363-370, doi:https://doi.org/10.1016/j.seppur.2007.01.039.
3. Bertone, E.; Chang, C.; Thiel, P.; O'Halloran, K. Analysis and modelling of powdered activated carbon dosing for taste and odour removal. *Water Research* **2018**, *139*, 321-328, doi:https://doi.org/10.1016/j.watres.2018.04.023.
4. Bong, T.; Kang, J.-K.; Yargeau, V.; Nam, H.-L.; Lee, S.-H.; Choi, J.-W.; Kim, S.-B.; Park, J.-A. Geosmin and 2-methylisoborneol adsorption using different carbon materials: Isotherm, kinetic, multiple linear regression, and deep neural network modeling using a real drinking water source. *Journal of Cleaner Production* **2021**, *314*, 127967, doi:https://doi.org/10.1016/j.jclepro.2021.127967.
5. Pochiraju, S.; Hoppe-Jones, C.; Weinrich, L.; Maalouf, S.; Adams, C. Treatability of 18 taste and odor compounds using powdered activated carbon in drinking water utilities. *AWWA Water Science* **2022**, *4*, e1289, doi:https://doi.org/10.1002/aws2.1289.
6. Alver, A.; Baştürk, E.; Altaş, L.; Işık, M. A solution of taste and odor problem with activated carbon adsorption in drinking water: detailed kinetics and isotherms. *Desalination and Water Treatment* **2022**, *252*, 300-318, doi:https://doi.org/10.5004/dwt.2022.28269.
7. Cerón-Vivas, A.; Villamizar León, M.P.; Cajigas, Á.A. Geosmin and 2-methylisoborneol removal in drinking water treatment. *Water Practice and Technology* **2022**, *18*, 159-167, doi:10.2166/wpt.2022.167.
8. Cui, Y.; Chen, X.; Pitakrattanawong, C.; Du, X.; Qiu, L.; Xu, H.; Chen, J.; Meng, S.; Fan, L.; Song, C. Adsorption efficiency of biochar produced by aquaculture by-products for removing geosmin in aquaculture environment. *Water Reuse* **2023**, *14*, 65-79, doi:10.2166/wrd.2023.129.
9. Antonopoulou, M.; Tzamaria, A.; Pedrosa, M.F.F.; Ribeiro, A.R.L.; Silva, A.M.T.; Kaloudis, T.; Hiskia, A.; Vlastos, D. Spirulina-based carbon materials as adsorbents for drinking water taste and odor control: Removal efficiency and assessment of cyto-genotoxic effects. *Science of The Total Environment* **2024**, *927*, 172227, doi:https://doi.org/10.1016/j.scitotenv.2024.172227.
10. Pirbazari, M.; Borow, H.S.; Craig, S.; Ravindran, V.; McGuire, M.J. Physical Chemical Characterization of Five Earthy-Musty-Smelling Compounds. *Water Science and Technology* **1992**, *25*, 81-88, doi:10.2166/wst.1992.0038.
11. Moretto, J.A.; Freitas, P.N.N.; Souza, J.P.; Oliveira, T.M.; Brites, I.; Pinto, E. Off-Flavors in Aquacultured Fish: Origins and Implications for Consumers. *Fishes* **2022**, *7*, 34.
